# Dexmedetomidine Ameliorates Myocardial Ischemia‐Reperfusion Injury by Inhibiting MDH2 Lactylation via Regulating Metabolic Reprogramming

**DOI:** 10.1002/advs.202409499

**Published:** 2024-10-28

**Authors:** Han She, Yi Hu, Guozhi Zhao, Yunxia Du, Yinyu Wu, Wei Chen, Yong Li, Yi Wang, Lei Tan, Yuanqun Zhou, Jie Zheng, Qinghui Li, Hong Yan, Qingxiang Mao, Deyu Zuo, Liangming Liu, Tao Li

**Affiliations:** ^1^ Department of Anesthesiology Daping Hospital Army Medical University Chongqing 400042 China; ^2^ Shock and Transfusion Department Daping Hospital Army Medical University Chongqing 400042 China; ^3^ Department of Urology Surgery The First Affiliated Hospital of Chongqing Medical University Chongqing 400016 China; ^4^ Department of Respiratory Disease Daping Hospital Army Medical University Chongqing 400042 China; ^5^ Department of Rehabilitation Medicine The First Affiliated Hospital of Chongqing University of Chinese Medicine Chongqing Traditional Chinese Medicine Hospital Chongqing 400021 China; ^6^ Department of Research and Development Chongqing Precision Medical Industry Technology Research Institute Chongqing 400000 China

**Keywords:** dexmedetomidine, ferroptosis, lactylation, metabolic reprogramming, myocardial ischemia‐reperfusion injury

## Abstract

Myocardial ischemia‐reperfusion injury (MIRI) significantly worsens the outcomes of patients with cardiovascular diseases. Dexmedetomidine (Dex) is recognized for its cardioprotective properties, but the related mechanisms, especially regarding metabolic reprogramming, have not been fully clarified. A total of 60 patients with heart valve disease are randomly assigned to Dex or control group. Blood samples are collected to analyze cardiac injury biomarkers and metabolomics. In vivo and vitro rat models of MIRI are utilized to assess the effects of Dex on cardiac function, lactate production, and mitochondrial function. It is found that postoperative CK‐MB and cTNT levels are significantly lower in the Dex group. Metabolomics reveals that Dex regulates metabolic reprogramming and reduces lactate level. In Dex‐treated rats, the myocardial infarction area is reduced, and myocardial contractility is improved. Dex inhibits glycolysis, reduces lactate, and improves mitochondrial function following MIRI. Lactylation proteomics identifies that Dex reduces the lactylation of Malate Dehydrogenase 2（MDH2), thus alleviating myocardial injury. Further studies reveal that MDH2 lactylation induces ferroptosis, leading to MIRI by impairing mitochondrial function. Mechanistic analyses reveal that Dex upregulates Nuclear Receptor Subfamily 3 Group C Member 1（NR3C1) phosphorylation, downregulates Pyruvate Dehydrogenase Kinase 4 (PDK4), and reduces lactate production and MDH2 lactylation. These findings provide new therapeutic targets and mechanisms for the treatment for MIRI.

## Graphical abstract

Dex reduces lactate levels and downregulates MDH2 lactylation to enhance mitochondrial function and prevent ferroptosis, ultimately alleviating myocardial ischemia‐reperfusion injury. This mechanism involves through Dex facilitating the phosphorylation and nuclear export of NR3C1, leading to the suppression of PDK4 and influencing metabolic reprogramming.

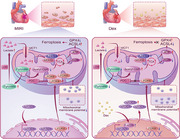

## 1 Introduction

Ischemic heart disease is a leading cause of death worldwide, accounting for 16% of total mortality. The number of deaths caused by this disease increased from 2 million in 2000 to 8.9 million in 2019.^[^ ^1^ ^]^ While reperfusion is essential for preventing cardiac function deterioration and improving clinical outcomes, restoring blood flow through thrombolysis or revascularization may result in severe myocardial ischemia/reperfusion injury (MIRI),^[^ ^1^ ^]^ which accounts for up to 50% of the final myocardial infarction area.^[^ ^2^ ^]^ Additionally, MIRI is a common complication in cardiopulmonary bypass and a critical factor leading to perioperative myocardial injury.^[^ ^3^ ^]^ The pathophysiology of MIRI is complex, involving oxidative stress, inflammatory responses, and various forms of cell death.^[^ ^4^, ^5^, ^6^ ^]^ Although significant theoretical breakthroughs have emerged in treating MIRI^[^ ^7^ ^]^over the past few decades, practical challenges remain in the applicability, safety, and efficacy of these treatments. Therefore, further research to identify measures for the prevention and treatment of MIRI is of significant clinical importance.

The heart is a critical organ with high energy consumption.^[^ ^8^ ^]^ Under normal physiological conditions, the heart relies on oxidative phosphorylation to produce ATP to meet its high energy demands.^[^ ^9^ ^]^ During myocardial ischemia‐reperfusion (I/R), the regular oxidative phosphorylation is inhibited, and glycolysis becomes the primary energy source. This metabolic shift leads to a rapid decrease in ATP production and an accumulation of lactate, which causes cellular damage.^[^ ^10^ ^]^ Studies have shown that lactate is not only a metabolic byproduct, but it also regulates gene expression through protein lactylation.^[^ ^11^ ^]^ Recent research indicated that lactate can limit mitochondrial oxidative phosphorylation via protein lactylation.^[^ ^12^ ^]^ However, the role and related mechanism of lactate as well as lactylation in the progression of MIRI remain unclear.

Dexmedetomidine (Dex) is a novel, highly selective α2‐adrenergic receptor agonist that has been widely used in clinical anesthesia and intensive care in recent years.^[^ ^13^, ^14^ ^]^ Its unique pharmacological properties extend beyond sedation, analgesia, and anxiolysis to provide significant cardiovascular protection.^[^ ^15^, ^16^ ^]^ For instance, perioperative administration of Dex has been shown to effectively reduce postoperative mortality and shorten hospital stay of patients after coronary artery bypass graft surgery.^[^ ^17^ ^]^ A high‐quality meta‐analysis involving 48 trials and 6273 participants also reported that perioperative use of Dex reduced short‐term mortality after cardiac surgery.^[^ ^18^ ^]^ Additionally, basic research indicates that Dex plays a crucial role in mitigating ischemia‐reperfusion injury, exhibiting antioxidative and anti‐inflammatory effects.^[^ ^15^, ^19^ ^]^ Our previous study has found that Dex can alleviate vascular leakage by inhibiting ferroptosis in vascular endothelial cells through metabolic reprogramming.^[^ ^20^ ^]^ However, it remains unclear whether Dex confers myocardial protection against I/R injury through metabolic remodeling.

In this study, we employed multi‐omics techniques to explore the role and mechanism of Dex in regulating myocardial metabolic reprogramming and alleviating MIRI. This research specifically focused on the role of lactate and lactylation, in hope to provide new insights for the prevention and treatment of MIRI.

## 2 Results

### 2.1 Dex reduced the Lactate Level of Cardiopulmonary Bypass Surgery Patients by Metabolic Reprogramming

**Figure 1:**
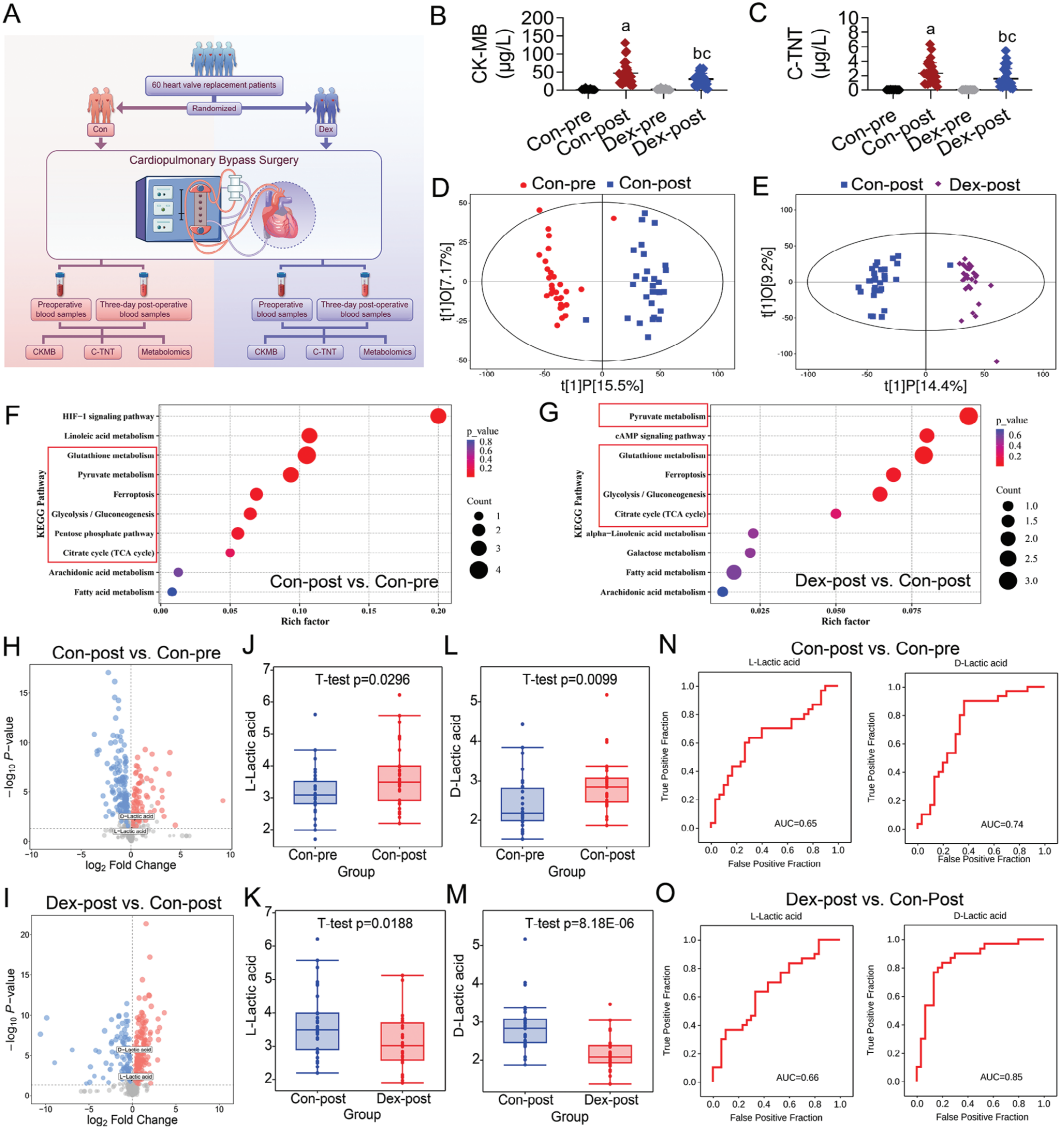
Dex regulated the lactate level of patients undergoing cardiopulmonary bypass surgery by metabolic reprogramming. A) Flowchart for collecting blood samples to measure cardiac injury biomarkers and metabolomics. B) Comparison of CK‐MB in the Con group and Dex group during the preoperative stage (pre) and the post‐operative stage (post) (*n* = 30 samples each group). C) Comparison of C‐TNT in the Con group and Dex group during the preoperative stage (pre) and the post‐operative stage (post) (*n* = 30 samples each group). D) Principal Component Analysis (PCA) of the difference in metabolic patterns between the Con‐pre and Con‐post groups. E) PCA of the difference in metabolic patterns between the Dex‐post and Con‐post groups. F,G) KEGG pathway enrichment analysis for differentially expressed (DE) metabolites. H,I) Volcano plot of DE‐metabolites, each dot represented a metabolite, blue indicated down‐regulated DE‐metabolites, red indicated up‐regulated, and gray indicated no significant difference. J,K) Box plots showing changes in levels of L‐lactic acid in different groups. L,M) Box plots showing changes in levels of D‐lactic acid in different groups. N,O) ROC curves evaluating the diagnostic capacity of L‐lactic acid and D‐lactic acid in different groups. a: p < 0.05, as compared with the Con‐pre group; b: p < 0.05, as compared with the Dex‐pre group; c: p < 0.05, as compared with the Con‐post group. Con‐pre: Con‐pre group; Con‐post: Con‐post group; Dex‐pre: Dex‐pre group; Dex‐post: Dex‐post group.

The temporarily interrupted blood supply for heart in cardiopulmonary bypass (CPB) surgery, could cause potential myocardial ischemia‐reperfusion injury (MIRI). Therefore, we included 60 patients diagnosed with heart valve disease and who underwent valve replacement surgery under CPB. The 60 patients were randomly divided into the Dex group and the Con group in a 1:1 ratio (The baseline information and the status of regular medications for the two groups of patients were shown in Table S2, Supporting Information). We collected venous blood both preoperatively (pre) and postoperatively (post) to measure the cardiac injury biomarkers CK‐MB and C‐TNT, as well as metabolomics (**Figure** **1**A). Results showed that the level of CK‐MB and C‐TNT in both Con and Dex group was low preoperatively (with no statistical difference between groups) but increased significantly postoperatively (Figure 1B,C). However, postoperative levels of CK‐MB and C‐TNT were significantly lower in the Dex group compared to the Con group (Figure 1B,C), suggesting that Dex effectively reduced MIRI.

Metabolomics was conducted to explore whether Dex conferred cardioprotective effects by modulating metabolic reprogramming. Principal Component Analysis (PCA) results indicated significant metabolic differences before and after surgery in both Con and Dex group (Figure 1D,E). We identified 232 differentially expressed (DE) metabolites between Con‐pre and Con‐post groups, enriched in Glycolysis/Gluconeogenesis, TCA cycle, Pyruvate metabolism, Ferroptosis, and Glutathione metabolism pathways. Similarly, there were 247 DE‐metabolites between Con‐post and Dex‐post groups, enriched in the same metabolic pathways (criteria for screening DE‐metabolites: VIP > 1 and P‐value < 0.05) (Figure 1F,G), suggesting that glycolysis activation and ferroptosis might be critical causes of MIRI, and the mechanism of Dex reducing MIRI might be related to regulating glycolysis and TCA cycle reprogramming and inhibiting ferroptosis. The metabolomics showed that the level of lactic acid significantly increased on the third day after surgery, while the postoperative lactic acid level in the Dex group was significantly lower than that in the Con group (Figure 1H,M). This finding indicated that Dex might influence lactic acid levels through metabolic reprogramming. In addition, the ROC curve results showed that lactic acid had a good discriminatory ability for preoperative and postoperative, as well as for Dex‐post and Con‐post. These results suggested that lactic acid might be a potential biomarker for MIRI (Figure 1N,O).

### 2.2 Dex Improved Mitochondrial Function and Reduced MIRI by Downregulating Lactate Level

**Figure 2:**
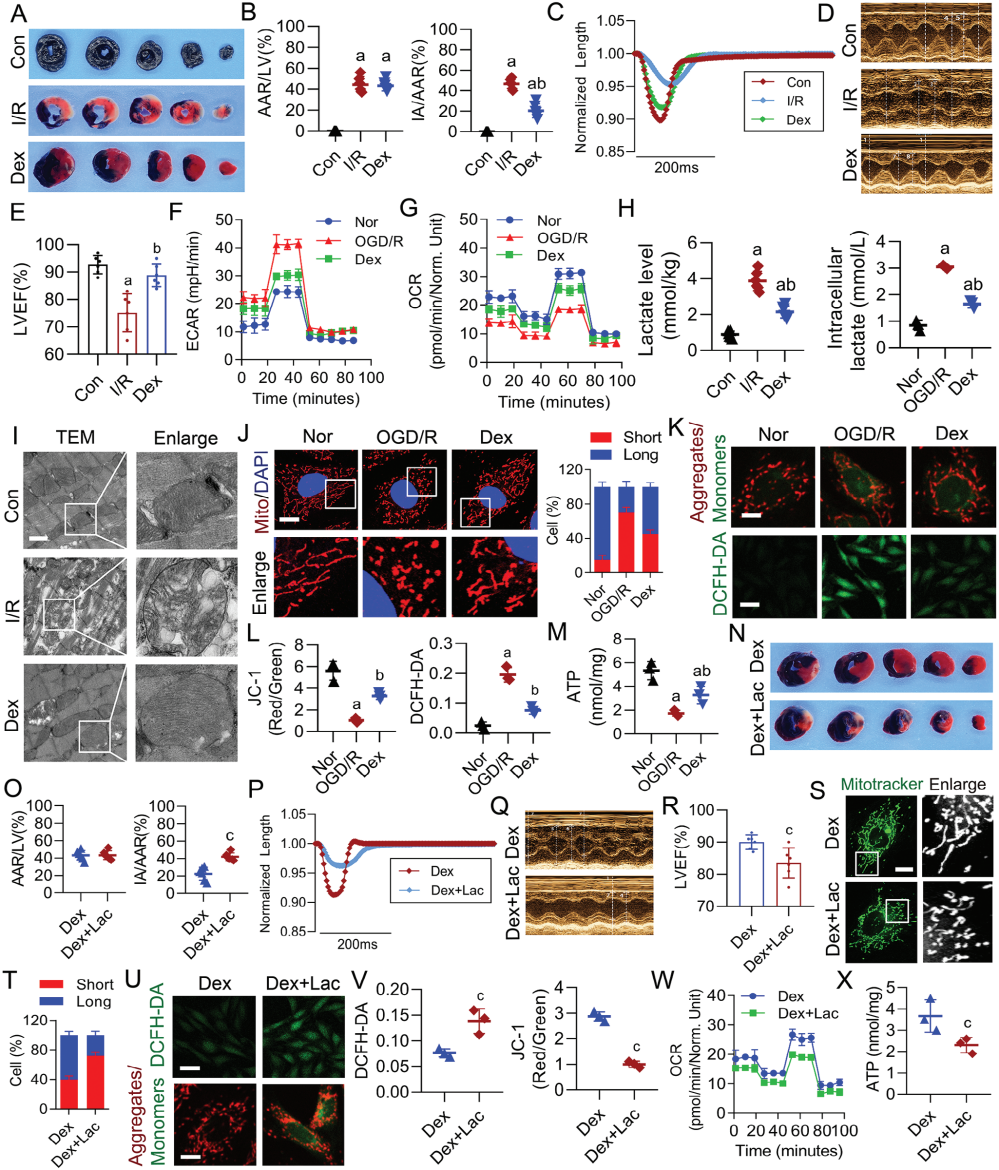
Dex enhanced mitochondrial function and mitigated MIRI by reducing lactate level. A,B) Evans Blue‐TTC staining was used to detect the impact of I/R and Dex on the myocardial infarction area in rats (*n* = 6 rats each group). The left ventricular area (LV), area at risk (AAR), and infarct area (IA) were calculated. AAR/LV (%) indicated ischemic region size, while IA/AAR (%) indicated infarcted region size. C) Cardiomyocyte contraction curves were used to reflect the impact of Dex on myocardial contractility in rats (*n* = 6 rats each group). D,E) Echocardiography was used to detect rats LVEF (*n* = 6 rats each group). F) ECAR was used to reflect the glycolysis rate (*n* = 3 independent experiments). G) Seahorse was used to detect mitochondrial OCR (*n* = 3 independent experiments). H) Lactate levels were measured in rats (*n* = 6 rats each group) and cells (*n* = 3 independent experiments). I) Transmission electron microscopy was used to observe the structure of myocardial mitochondria (bar = 1µm) (*n* = 6 rats each group). J) Confocal microscopy was used to observe mitochondrial morphology of H9c2 cells (each group randomly selected 30 cells and the mitochondrial morphology was blindly scored and classified into two categories: Long (>6µm), Short (≤3µm) (bar = 15µm) (*n* = 3 independent experiments)). K,L) JC‐1 and DCFH‐DA were used to detect mitochondrial membrane potential (bar = 15µm) and ROS (bar = 50µm) respectively (*n* = 3 independent experiments). M) The effect of Dex on ATP levels in OGD/R‐treated cells (*n* = 3 independent experiments). N,O) Evans Blue‐TTC staining was used to detect the effect of exogenous lactate on myocardial infarction area (*n* = 6 rats each group). The effect of exogenous lactate on P) cardiomyocytes contractility and Q,R) LVEF (*n* = 6 rats each group). S,T) The effect of exogenous lactate on mitochondrial morphology (bar = 15µm) (*n* = 3 independent experiments). U,V) The effect of exogenous lactate on ROS (bar = 50µm) and mitochondrial membrane potential of H9c2 cells (bar = 15µm) (*n* = 3 independent experiments). The effect of exogenous lactate on W) mitochondrial OCR and X) ATP levels (*n* = 3 independent experiments) of H9c2 cells. a: p < 0.05, as compared with the Con or Nor group; b: p < 0.05, as compared with the I/R or OGD/R group; c: p < 0.05, as compared with the Dex group. Con: control group; Nor: normal group; I/R: I/R group; OGD/R: OGD/R group; Dex: Dex‐treated I/R or OGD/R group; Dex + Lac: Dex + Lac treated I/R or OGD/R group.

We constructed a rat model of MIRI and observed the protective effects of Dex. The results showed that compared to the Con group, I/R injury significantly impaired cardiac function, manifesting as a significant increase in myocardial infarction area (**Figure** **2**A,B), a marked decrease in myocardial contractility, and a reduction in LVEF (Figure 2C–E). However, compared to the I/R group, Dex treatment effectively alleviated MIRI, as evidenced by a reduction in infarction area, increased myocardial contractility, and elevated LVEF (Figure 2A–E). Additionally, we used OGD/R to establish an I/R model at the cellular level and analyzed the effects of OGD/R and Dex treatment on metabolic reprogramming in cardiomyocytes. The results showed that OGD/R led to significant increases in glycolysis rate and reductions in mitochondrial oxygen consumption rate (OCR), which were all relieved by Dex treatment (Figure 2F,G). Additionally, we found that MIRI increased the lactate level in cardiomyocytes, whereas Dex effectively inhibited lactate production (Figure 2H).

Furthermore, we examined the effects of OGD/R and Dex treatment on the structure and function of myocardial mitochondria. Transmission electron microscopy and confocal microscopy results showed that compared to the Con (Nor) group, I/R (OGD/R) caused abnormal mitochondrial structures, characterized by increased mitochondrial vacuolization, cristae disruption, and mitochondrial fragmentation, whereas Dex effectively improved mitochondrial morphology and reduced mitochondrial fragmentation (Figure 2I,J). Moreover, OGD/R induced mitochondrial dysfunction in cardiomyocytes, manifested by decreased mitochondrial membrane potential, increased reactive oxygen species (ROS) production, and reduced ATP levels, which were all improved by Dex treatment (Figure 2K–M). These results suggested that the protective effects of Dex after MIRI might be related to regulating metabolic reprogramming, inhibiting lactate production, and improving mitochondrial function.

To further verify the role of lactate in this process, we pretreated cells and rats by adding exogenous lactate (sodium lactate, Lac) to directly upregulate lactate levels.^[^ ^21^ ^]^ The results showed that lactate concentrations ≥10 mm significantly reduced the viability of cardiomyocytes (Figure S1, Supporting Information). Additionally, we found that lactate administration effectively counteracted the protective effects of Dex on myocardial injury in I/R rats. Compared to the Dex group, the Dex + Lac showed a significant increase in infarct size, reduced myocardial contractility, and lower LVEF (Figure 2N–R). Similarly, lactate administration counteracted the protective effects of Dex on the structure and function of mitochondria in OGD/R‐treated cells. Compared to the Dex group, the Dex + Lac increased mitochondrial fragmentation, decreased mitochondrial membrane potential, elevated ROS production, inhibited mitochondrial OCR, and reduced ATP production (Figure 2S–X). These results indicated Dex could improve mitochondrial function and reduce MIRI by downregulating lactate level.

### 2.3 Dex Downregulated the Lactylation of MDH2

**Figure 3:**
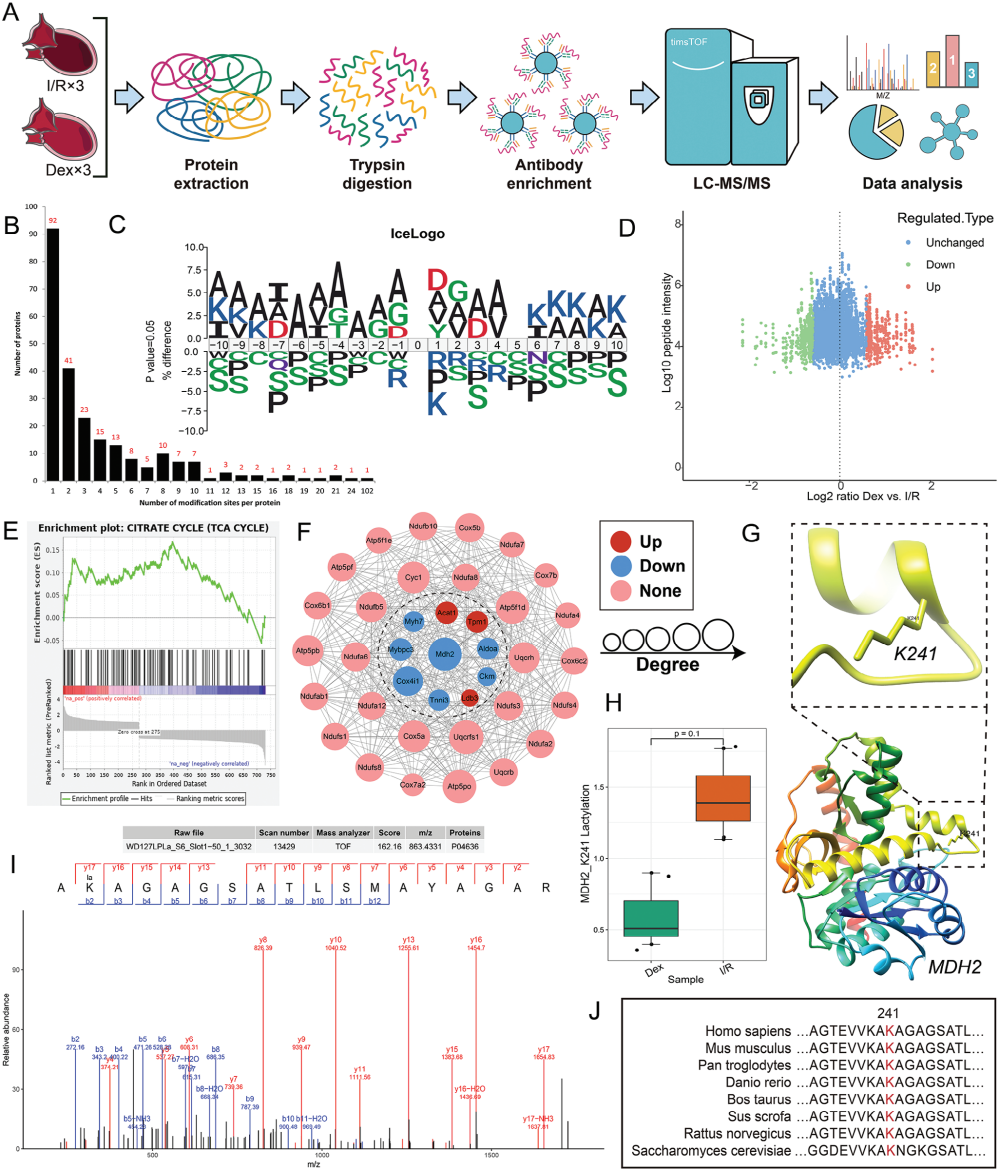
Lactylation proteomics indicated that Dex reduced the lactylation of MDH2. A) Workflow of the strategy for lactylation proteomics analysis. B) Statistical distribution of lactylation sites. C) Icelogo representation displaying flanking sequence preferences for all Kla sites. D) Scatterplot illustrating the quantification of Kla sites in relation to peptide intensities. E) Pathway enrichment analysis via GSEA. F) Protein‐protein interaction network of Kla proteins (Top10) based on the STRING database. G) Crystal structure of MDH2. H) Relative expression of MDH2 K241 lactylation between the Dex and I/R groups. I) MS spectrum of the Kla 241 site. J) Evolutionary conservation of MDH2 K241 site. The sequences of MDH2 in 8 species were aligned with K241 highlighted in red. Dex: Dex group; I/R: I/R group.

Lactate regulated cell functions by affecting protein lysine lactylation (Kla). Therefore, we collected myocardial tissues from I/R and Dex rats and conducted lactylation proteomics (**Figure** **3**A). A total of 1026 lactylation sites across 238 proteins were identified, among them, 731 lactylation sites from 167 proteins were quantified. The number of Kla sites per protein is shown in Figure 3B. Next, we used the iceLogo tool to analyze the amino acids surrounding the identified Kla sites against all human background sequences. Significant enrichment Alanine and Aspartate were found at the −1 and +1 positions of the Kla sites (Figure 3C). A volcano plot of the differential analysis showed that, compared to the I/R group, 446 lactylation‐modified peptides were significantly upregulated, but 554 lactylation‐modified peptides were significantly downregulated in the Dex group (Figure 3D). GSEA pathway enrichment analysis of the differential proteins revealed that the TCA cycle pathway was significantly enriched (Figure 3E), suggesting that Dex might regulate the lactylation of TCA cycle‐related proteins. Using Protein‐Protein Interaction (PPI) interaction analysis to score and screen key proteins, we found that malate dehydrogenase 2 (MDH2) was the hub protein among the identified Kla proteins (Figure 3F). MDH2 is a critical enzyme located in the mitochondrial matrix and plays an essential role in the TCA cycle.^[^ ^22^ ^]^ Immunofluorescence and WB analyses were used to confirm that MDH2 is localized in the mitochondria (Figure S2A,B, Supporting Information). Lactylation proteomics analysis revealed that the Lys241 (K241) site of MDH2 was significantly downregulated in the Dex group compared to the I/R group (Figure 3G–I). MDH2 K241 was found to be highly conserved among different species (Figure 3J). These results indicated that Dex could downregulate the lactylation of MDH2 in MIRI.

### 2.4 Dex Attenuated MIRI by Improving Mitochondrial Function Through Downregulating MDH2 K241 Lactylation

**Figure 4:**
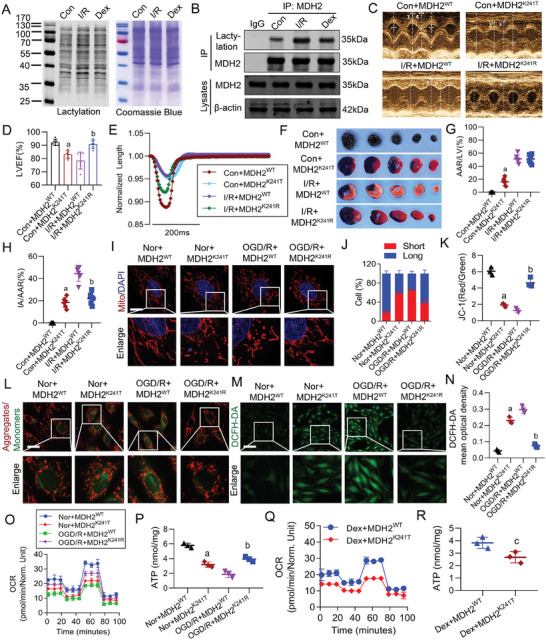
Dex reduced myocardial I/R injury by enhancing mitochondrial function via downregulating MDH2 K241 lactylation. A) The effect of Dex on the myocardial lactylation level in I/R rats was detected by WB (*n* = 3 independent experiments). B) The lactylation level of MDH2 was detected by immunoprecipitation (*n* = 3 independent experiments). C,D) Echocardiography was used to detect the impact of the MDH2 lactylation regulation on the LVEF in rats (*n* = 6 rats each group). E) Cardiomyocyte contraction curves was used to reflect the impact of the MDH2 lactylation regulation on the myocardial contractility in rats (*n* = 6 rats each group). F–H) Evans blue‐TTC staining was used to detect the impact of the MDH2 lactylation regulation on the myocardial infarction area in rats (*n* = 6 rats each group). I,J) Changes in mitochondrial morphology in cells with the MDH2 K241 mutation were observed, 30 cells were randomly selected in each group and the mitochondrial morphology was blindly scored and classified into two categories: Long (>6µm) and Short (≤3µm) (bar = 10µm) (*n* = 3 independent experiments). K,L) Changes in mitochondrial membrane potential in cells with the MDH2 K241 mutation were observed, with membrane potential levels reflected by calculating the red/green fluorescence intensity via ImageJ (bar = 40µm) (*n* = 3 independent experiments). M,N) Changes in ROS levels in cells with the MDH2 K241 mutation were observed, with ROS levels reflected by calculating the green fluorescence intensity (bar = 150µm) (*n* = 3 independent experiments). O) Changes in mitochondrial OCR and P) ATP levels in cells with the MDH2 K241 mutation were observed (*n* = 3 independent experiments). Q) Effects of MDH2 K241T on mitochondrial OCR and R) ATP levels in Dex‐treated OGD/R cells were observed (*n* = 3 independent experiments). a: p < 0.05, as compared with the Con + MDH2^WT^ or Nor + MDH2^WT^ group; b: p < 0.05, as compared with the I/R + MDH2^WT^ or OGD/R + MDH2^WT^ group. Con: control group; I/R: I/R group; Dex: Dex‐treated I/R group; Con + MDH2^WT^: Control + MDH2^WT^ group; Con + MDH2^K241T^: Control + MDH2^K241T^ group; I/R + MDH2^WT^: I/R + MDH2^WT^ group; I/R + MDH2^K241R^: I/R + MDH2^K241R^ group; Nor + MDH2^WT^: Normal + MDH2^WT^ group; Nor + MDH2^K241T^: Normal + MDH2^K241T^ group; OGD/R + MDH2^WT^: OGD/R + MDH2^WT^ group; OGD/R + MDH2^K241R^: OGD/R + MDH2^K241R^ group; Dex + MDH2^WT^: Dex + MDH2^WT^ + OGD/R group: Dex + MDH2^K241T^: Dex + MDH2^K241T^ + OGD/R group.

We validated the results of lactylation proteomics by collecting myocardial tissues and using the pan‐antibody to detect the total protein lactylation levels. The results indicated that MIRI led to an upregulation of lactylation in cardiomyocytes, which was downregulated by Dex (**Figure** **4**A). Furthermore, we assessed the lactylation levels of MDH2 using immunoprecipitation, and found that MIRI significantly increased MDH2 lactylation, which was inhibited by Dex treatment, being consistent with the results of lactylation proteomics (Figure 4B). Since MDH2 is primarily localized in the mitochondria, we employed the mitochondrial lactate probe, Fila‐Mit^[^ ^23^ ^]^ to assess changes in mitochondrial lactate levels under different conditions. Our results demonstrated that OGD/R significantly increased mitochondrial lactate level in H9c2 cells, whereas Dex effectively reduced the level of mitochondrial lactate. Furthermore, adding lactate to the Dex group increased mitochondrial lactate level, while knocking down MCT1 effectively decreased mitochondrial lactate level (Figure S2C–E, Supporting Information). To clarify the function of lactylation at the MDH2 K241 site, we constructed MDH2 point‐mutant rats and cells in vivo and in vitro using adeno‐associated virus and lentivirus, respectively (Figure S3, Supporting Information). Previous studies showed that mutating lysine (K) to threonine (T) can be used to simulate lactylation, while mutating lysine (K) to arginine (R) can be used to simulate delactylation.^[^ ^24^, ^25^, ^26^, ^27^ ^]^ Therefore, we simulated MDH2 K241 lactylation and delactylation using MDH2^K241T^ and MDH2^K241R^. The results showed that simulating MDH2 K241 lactylation led to myocardial injury, manifested by decreased LVEF, reduced myocardial contractility, and increased myocardial infarction area (Figure 4C–H). Conversely, simulating K241 delactylation improved the cardiac function of I/R rats, as evidenced by improved LVEF, enhanced myocardial contractility, and reduced myocardial infarction area (Figure 4C–H).

Additionally, we investigated the impact of MDH2 K241 lactylation on the structure and function of myocardial mitochondria. Simulating MDH2 K241 lactylation resulted in mitochondrial damage, indicated by increased mitochondrial fragmentation, reduced mitochondrial membrane potential, elevated ROS production, and decreased mitochondrial OCR and ATP levels (Figure 4I–P). In contrast, simulating MDH2 K241 delactylation improved the structure and function of mitochondria in OGD/R‐treated cardiomyocytes cells, as it enhanced mitochondrial morphology, increased membrane potential, inhibited ROS production, and elevated mitochondrial OCR and ATP levels (Figure 4I–P). Moreover, simulating MDH2 K241 lactylation hindered the protective effects of Dex on mitochondrial OCR and ATP production in OGD/R cells (Figure 4Q,R). These findings suggested that Dex attenuated MIRI by improving mitochondrial function via downregulating MDH2 K241 lactylation.

### 2.5 Dex Improved Mitochondrial Function by Regulating MDH2 K241 Lactylation to Inhibit Ferroptosis

**Figure 5:**
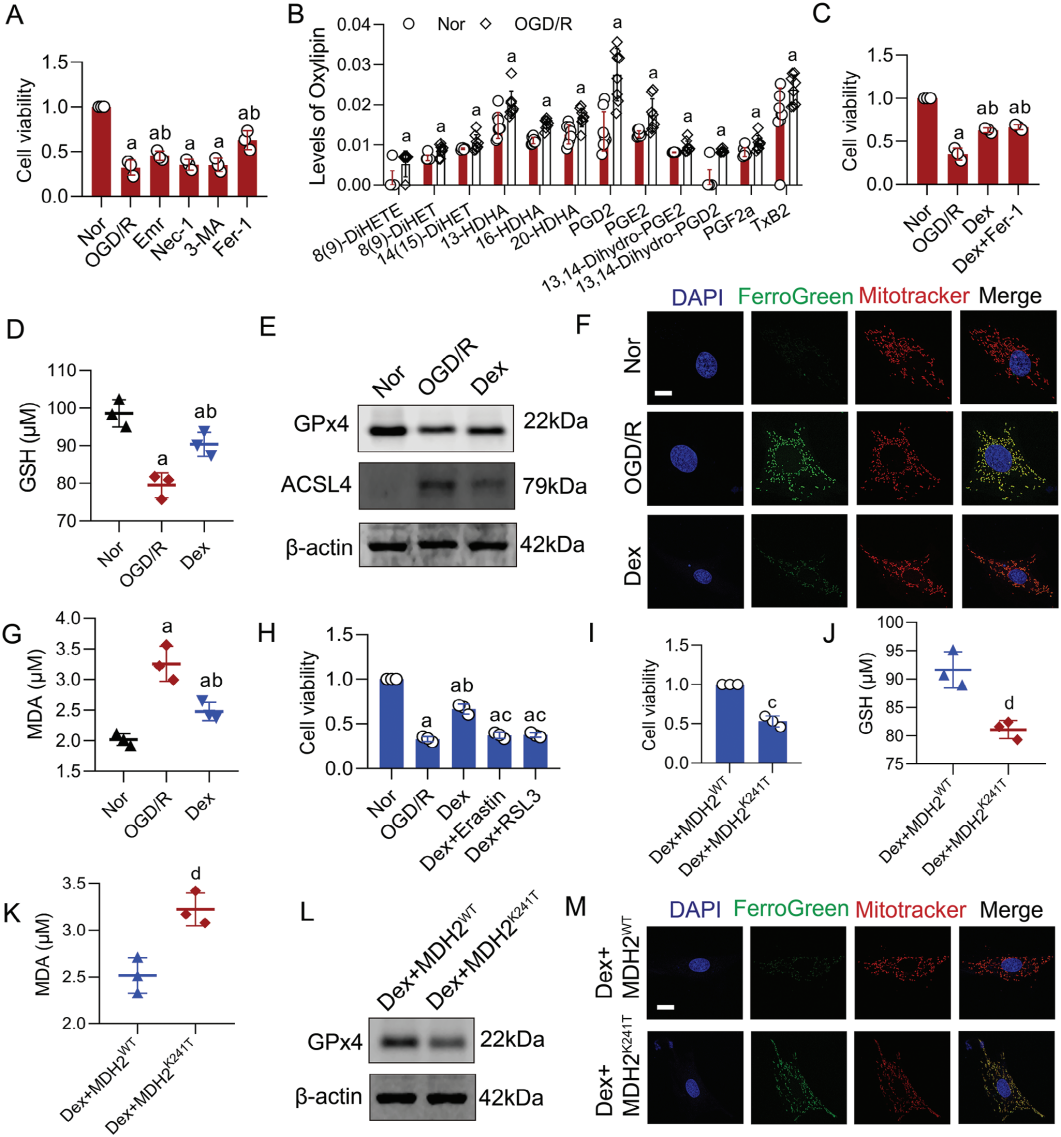
Dex enhanced mitochondrial function by modulating MDH2 K241 lactylation to prevent ferroptosis. A) The effect of different inhibitors on the viability of OGD/R‐treated cells was detected using a CCK8 assay (*n* = 3 independent experiments). B) The effect of OGD/R on oxylipin levels was detected using lipidomics analysis (*n* = 6 independent experiments). C) The effect of Dex on OGD/R‐treated cell viability was detected (*n* = 3 independent experiments). D) The effect of Dex on GSH levels in OGD/R‐treated cells was analyzed (*n* = 3 independent experiments). E) The effect of Dex on GPX4 and ACSL4 levels in OGD/R‐treated cells was detected using WB (*n* = 3 independent experiments). F) The effect of Dex on mitochondrial ferrous ion levels in OGD/R‐treated cells was measured (bar = 15µm) (*n* = 3 independent experiments). G) The effect of Dex on MDA levels in OGD/R‐treated cells was examined (*n* = 3 independent experiments). H) The effect of ferroptosis inducers Erastin and RSL3 on the viability of Dex‐treated H9c2 cells was detected by CCK8 (*n* = 3 independent experiments). I) The effect of MDH2 K241T mutation on the viability of Dex‐treated H9c2 cells was detected (*n* = 3 independent experiments). J) The effect of MDH2 K241T mutation on GSH levels in Dex‐treated H9c2 cells was analyzed (*n* = 3 independent experiments). K) The effect of MDH2 K241T mutation on MDA levels in Dex‐treated H9c2 cells was examined (*n* = 3 independent experiments). L) The effect of MDH2 K241T mutation on GPX4 levels in Dex‐treated H9c2 cells was detected by WB (*n* = 3 independent experiments). M) The effect of MDH2 K241T mutation on mitochondrial ferrous ion levels in Dex‐treated H9c2 cells was measured (bar = 15µm) (*n* = 3 independent experiments). a: p < 0.05, as compared with the Normal group; b: p < 0.05, as compared with the OGD/R group; c: p < 0.05, as compared with the Dex group; d: p < 0.05, as compared with the Dex + MDH2^WT^ group. Nor: normal group; OGD/R: OGD/R group; Dex: Dex‐treated OGD/R group; Dex + Fer‐1: Dex + Fer‐1 + OGD/R group; Dex + Erastin: Dex + Erastin + OGD/R group; Dex + RSL3: Dex + RSL3 + OGD/R group; Dex + MDH2^WT^: Dex + MDH2^WT^ + OGD/R group: Dex + MDH2^K241T^: Dex + MDH2^K241T^ + OGD/R group.

Based on the close relationship between mitochondrial function and cell death,^[^ ^28^ ^]^ we further investigated whether Dex alleviated I/R injury by inhibiting myocardial cell death through improving mitochondrial function. Through metabolic pathway enrichment analysis of patients undergoing cardiopulmonary bypass surgery, we discovered that ferroptosis and glutathione pathways were significantly enriched, suggesting that ferroptosis might play a crucial role of Dex in improving myocardial I/R injury (Figure 1F,G). The CCK8 results revealed that Fer‐1 (an inhibitor of ferroptosis) and Emr (an inhibitor of apoptosis), but not Nec‐1 (an inhibitor of necroptosis) or 3‐MA (an inhibitor of autophagy), significantly improved myocardial cell viability after OGD/R treatment, with Fer‐1 showing a more significant effect (**Figure** **5**A), indicating that ferroptosis might be a key cause of myocardial I/R injury. Given the close relationship between ferroptosis and lipid peroxidation,^[^ ^29^ ^]^ we employed lipid omics to detect changes in oxylipins in cardiomyocytes treated with OGD/R. The results showed a significant increase in various oxylipins in cardiomyocytes after OGD/R treatment, further confirming the important role of ferroptosis (Figure 5B). Meanwhile, we found that compared to the OGD/R group, Dex significantly improved the viability of cardiomyocytes, and the combined effect of Dex + Fer‐1 on cell viability was not significantly different from the Dex group (Figure 5C), suggesting that Dex may primarily provide protection against MIRI by reducing ferroptosis in myocardial cells.

Subsequently, we examined multiple ferroptosis‐related indicators and found that OGD/R significantly reduced GSH and GPX4 levels, and markedly increased mitochondrial ferrous ion and MDA levels. Dex significantly alleviated ferroptosis, as it increased GSH and GPX4 levels, and decreased mitochondrial ferrous ion and MDA levels (Figure 5D–G). Additionally, ferroptosis inducers Erastin and RSL3 effectively counteracted Dex's benefits on cell viability following OGD/R (Figure 5H). These results suggested that Dex might exert protective effects against I/R injury primarily by improving mitochondrial function and reducing ferroptosis in cardiomyocytes. Furthermore, we examined the impact of the MDH2 K241T mutation on Dex's reduction of myocardial ferroptosis. The results showed that the MDH2 K241T mutation effectively counteracted Dex's inhibition on ferroptosis in OGD/R‐treated cells, resulting in decreased cell viability, reduced GSH levels, increased MDA levels, decreased GPX4 levels, and elevated mitochondrial ferrous ion levels in the MDH2 K241T mutation group (Figure 5I–M). These findings suggested that Dex might alleviate ferroptosis through improving mitochondrial function by regulating MDH2 lactylation.

### 2.6 Dex Downregulated PDK4 to Inhibit Lactate Production and Alleviate Ferroptosis

**Figure 6:**
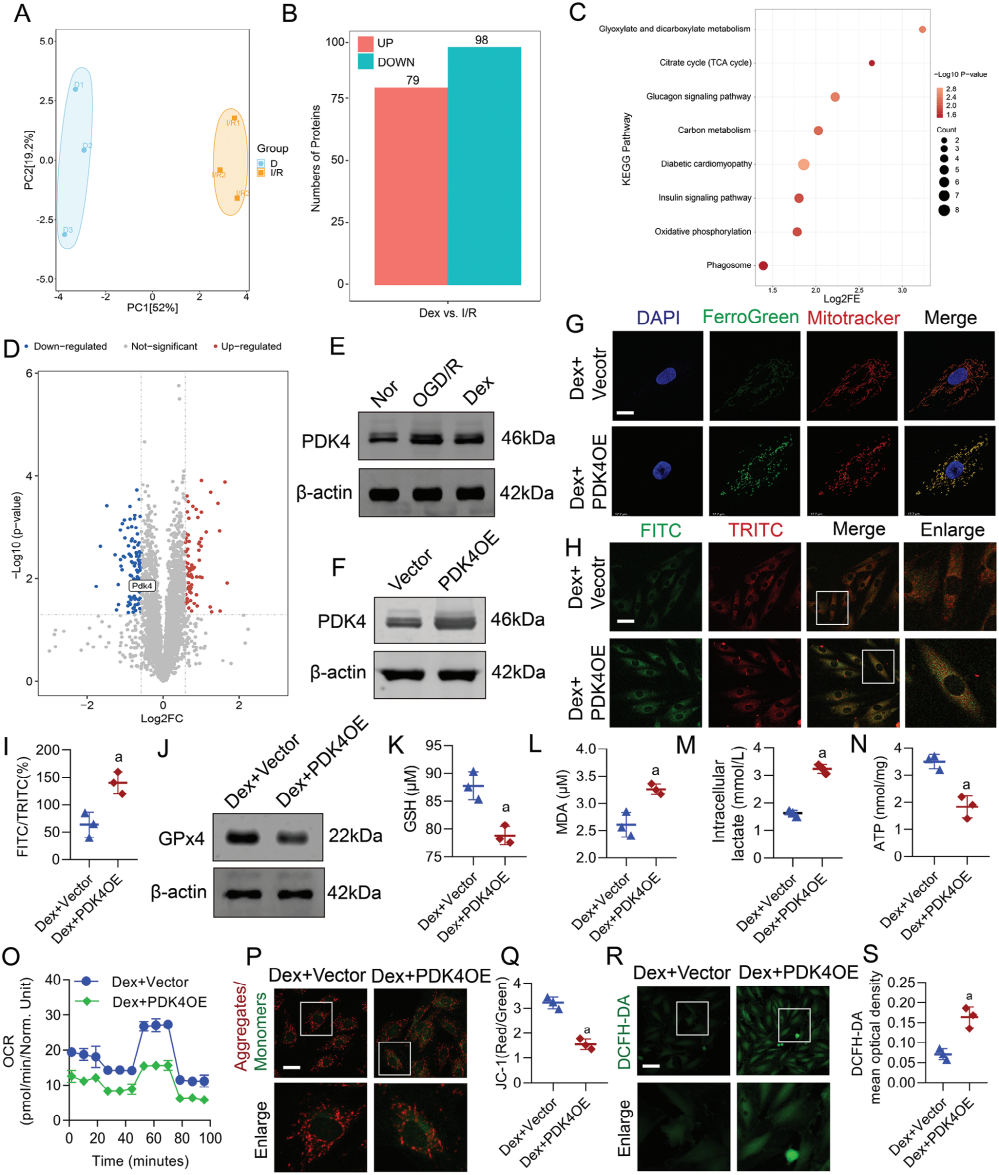
Dex downregulated PDK4 to reduce lactate production and alleviate ferroptosis. A) PCA analysis of protein expression differences between the Dex group and the I/R group. B) Differential analysis of Dex vs. I/R showed 98 upregulated proteins and 79 downregulated proteins, with red indicating upregulation and blue indicating downregulation. C) Pathway enrichment analysis of differentially expressed (DE) proteins: circle size represents the number of enriched proteins, and deeper red indicates a more significant P‐value. D) Volcano plot of DE‐proteins (Dex vs I/R), with blue indicating downregulated proteins, red indicating upregulated proteins, and gray indicating not significant. E) Western blot analysis of the effect of Dex on PDK4 in H9c2 cells (*n* = 3 independent experiments). F) Western blot analysis of PDK4 overexpression (OE) efficiency (*n* = 3 independent experiments). G) PDK4 OE increased mitochondrial ferrous ion levels in H9c2 cells (bar = 15µm) (*n* = 3 independent experiments). H) PDK4 OE increased lipid peroxidation in H9c2 cells (bar = 40µm) (*n* = 3 independent experiments). I) Lipid peroxidation statistical results, reflected by FITC/TRITC calculated with ImageJ (*n* = 3 independent experiments). J) Western blot analysis of the impact of PDK4 OE on GPX4 levels (*n* = 3 independent experiments). Impact of PDK4 OE on K) GSH, L) MDA, and M) intracellular lactate levels in H9c2 cells (*n* = 3 independent experiments). N) Impact of PDK4 OE on ATP levels in H9c2 cells (*n* = 3 independent experiments). O) Impact of PDK4 OE on OCR in H9c2 cells (*n* = 3 independent experiments). P,Q) Impact of PDK4 OE on mitochondrial membrane potential in H9c2 cells (bar = 40µm) (*n* = 3 independent experiments). R,S) Impact of PDK4 OE on ROS generation in H9c2 cells (bar = 60µm) (*n* = 3 independent experiments). a: p < 0.05, as compared with the Dex + Vector group. Nor: normal group; OGD/R: OGD/R group; Dex: Dex‐treated OGD/R group; Vector: Vector group; PDK4OE: PDK4OE group; Dex + Vector: Dex + Vector + OGD/R group; Dex + PDK4OE: Dex + PDK4OE + OGD/R group.

We further investigated the mechanism by which Dex regulated lactate levels. PCA revealed significant differences in protein expression between the Dex and I/R group, identifying 177 differentially expressed proteins—98 upregulated and 79 downregulated (Dex vs I/R). The criteria for differential protein screening included unique peptide ≥1, fold change ≥ 1.5 or ≤0.67, and P‐value < 0.05 (**Figure** **6**A,B). Pathway enrichment analysis using the KEGG database indicated that these proteins were primarily enriched in pathways such as the TCA cycle and oxidative phosphorylation, aligning with our previous results (Figure 6C). Notably, the protein pyruvate dehydrogenase kinase 4 (PDK4), which was closely related to lactate metabolism, was significantly downregulated in the Dex group (Figure 6D).

Previous studies showed that PDK4 promoted lactate production by inhibiting the activity of the pyruvate dehydrogenase complex (PDC).^[^ ^30^ ^]^ Western blot analysis demonstrated that OGD/R significantly upregulated PDK4 levels compared to normal group, while Dex effectively inhibited PDK4 expression (Figure 6E), consistent with our proteomic results. To determine whether Dex inhibits lactate production and ferroptosis by downregulating PDK4, we utilized the adenovirus to overexpress PDK4 (Figure 6F) and examined whether PDK4 overexpression (PDK4 OE) could counteract the inhibitory effects of Dex on lactate production and ferroptosis under OGD/R. The results showed that PDK4 OE significantly increased mitochondrial ferrous ion levels in H9c2 cells and promoted lipid peroxidation compared to the Dex group (Figure 6G–I). Additionally, PDK4 OE downregulated GPX4 and reduced GSH levels, and upregulated MDA levels in Dex‐treated H9c2 cells (Figure 6J–L). PDK4 OE also counteracted the inhibitory effect of Dex on lactate production and MDH2 lactylation (Figure 6M; Figure S4A, Supporting Information). Moreover, we explored whether PDK4 OE could negate the protective effects of Dex on mitochondrial function in H9c2 cells. The findings indicated that PDK4 OE significantly impaired mitochondrial function in Dex‐treated H9c2 cells, characterized by inhibited ATP production, reduced OCR and mitochondrial membrane potential, and increased ROS generation (Figure 6N–S).

### 2.7 Dex Downregulated PDK4 by Upregulating NR3C1 Phosphorylation

**Figure 7:**
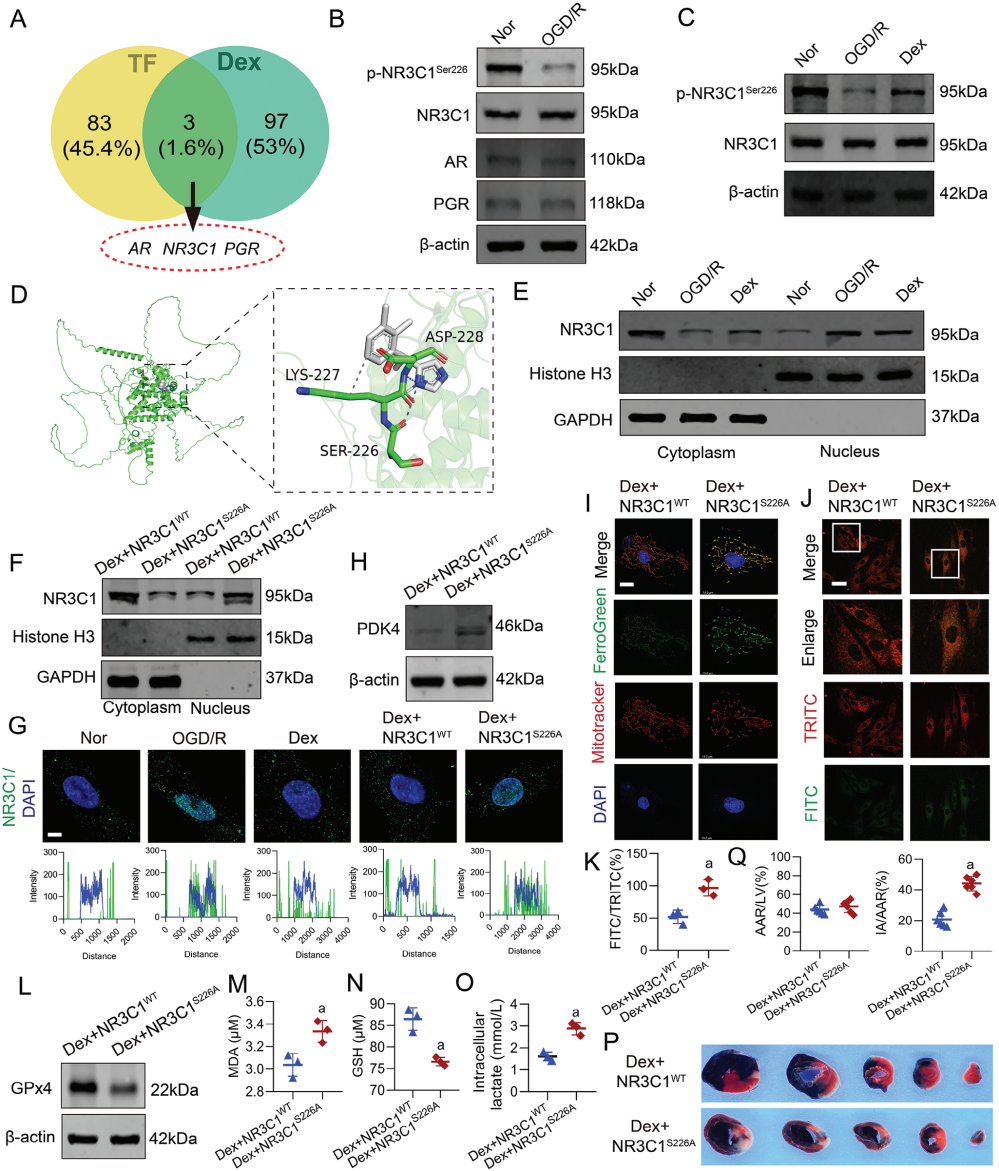
Dex enhanced NR3C1 phosphorylation leading to PDK4 downregulation. A) Venn analysis was used to screen for transcription factors regulated PDK4 that are affected by Dex. B) WB was used to detect the effects of OGD/R on the phosphorylation of NR3C1 and the expression of NR3C1, AR, and PGR (*n* = 3 independent experiments). C) WB was used to detect the effect of Dex on the phosphorylation of NR3C1 (*n* = 3 independent experiments). D) Molecular docking analysis of the binding of Dex to the phosphorylation site Ser226 of NR3C1. E) WB analysis of the effect of Dex on the distribution of NR3C1 in the cytoplasm and nucleus, with Histone H3 and GAPDH as internal references for nuclear and cytoplasmic proteins respectively (*n* = 3 independent experiments). F) The effect of NR3C1^S226A^ mutation on the distribution of NR3C1 in the cytoplasm and nucleus (*n* = 3 independent experiments). G) Immunofluorescence analysis of the changes in colocalization of NR3C1 with the nucleus under different treatment conditions (bar = 5µm) (*n* = 3 independent experiments). H) WB was used to detect the effect of NR3C1^S226A^ mutation on PDK4 expression (*n* = 3 independent experiments). I) Immunofluorescence was used to detect the effect of NR3C1^S226A^ mutation on the level of mitochondrial iron ions in H9c2 cells (bar = 10µm) (*n* = 3 independent experiments). J,K) Effect of NR3C1^S226A^ mutation on lipid peroxidation in H9c2 cells (bar = 50µm) (*n* = 3 independent experiments). L) WB was used to detect the effect of NR3C1^S226A^ mutation on GPX4 expression (*n* = 3 independent experiments). Effect of NR3C1^S226A^ mutation on M) MDA levels, N) GSH levels, and O) intracellular lactate levels in H9c2 cells (*n* = 3 independent experiments). P,Q) Evans blue‐TTC staining was used to detect the effect of NR3C1^S226A^ mutation on myocardial I/R injury (*n* = 6 rats each group). a: p < 0.05, compared with the Dex + NR3C1^WT^ group. Nor: normal group; OGD/R: OGD/R group; Dex: Dex‐treated OGD/R group; Dex + NR3C1^WT^: Dex + NR3C1^WT^ + OGD/R (I/R) group; Dex + NR3C1^S226A^: Dex + NR3C1^S226A^ + OGD/R (I/R) group.

To investigate the mechanism by which Dex downregulated PDK4, we utilized the hTFtarget database (https://guolab.wchscu.cn/GuoLab/) to identify 86 transcription factors that regulated PDK4. Subsequently, we performed a VENN analysis with 100 targets of Dex obtained from the Swiss Target Prediction database (http://swisstargetprediction.ch/), and found three intersecting genes: nuclear receptor subfamily 3 group C member 1(NR3C1), androgen receptor (AR), and progesterone receptor (PGR) (**Figure** **7**A). The Western blot results indicated no significant changes in the expression of NR3C1, AR, and PGR between the OGD/R and the normal group, but the phosphorylation level of NR3C1 was significantly reduced in the OGD/R group. (Figure 7B). Dex significantly upregulated the phosphorylation level of NR3C1 (Figure 7C). Molecular docking results also revealed that Dex directly interacted with the Ser226 site of NR3C1, forming hydrogen bonds (Figure 7D).

NR3C1 was typically located in the cytoplasm in an inactive state. Upon activation, NR3C1 dissociated from its chaperone protein complex and translocated to the nucleus, thereby regulating the transcription of target genes.^[^ ^31^, ^32^ ^]^ The phosphorylation level significantly influenced NR3C1's transcriptional activity and subcellular localization.^[^ ^33^ ^]^ To verify the hypothesis that Dex affected PDK4 expression by altering the phosphorylation and nuclear localization of NR3C1, we separated nuclear and cytoplasmic proteins and used Western blot to detect the expression of related protein. Results showed that OGD/R significantly upregulated NR3C1 expression in the nucleus but downregulated it in the cytoplasm. Dex treatment increased cytoplasmic NR3C1 levels and decreased nuclear NR3C1 expression (Figure 7E). Mutation at the Ser226 site (S226A), which mimicked dephosphorylation, counteracted Dex's regulatory effect on NR3C1 nuclear localization (Figure 7F). Immunofluorescence colocalization results corroborated the Western blot findings (Figure 7G). The NR3C1 S226A mutation effectively counteracted Dex's inhibitory effect on PDK4 expression (Figure 7H). We further examined whether inhibiting NR3C1 phosphorylation could counteract the effect of Dex on of I/R‐induced ferroptosis and lactate production in cardiomyocytes. Results revealed that inhibiting phosphorylation of NR3C1 by S226A mutation increased mitochondrial ferrous ion and lipid peroxidation, MDA, intracellular lactate level, and MDH2 lactylation, but decreased GPX4 and GSH levels in Dex‐treated cardiomyocytes (Figure 7I–O; Figure S4B, Supporting Information). Meanwhile, utilizing AAV to inhibit phosphorylation of NR3C1 in rats weakened the protective effect of Dex on cardiomyocytes in I/R rats (Figure 7P,Q).

## 3 Discussion

**Figure 8:**
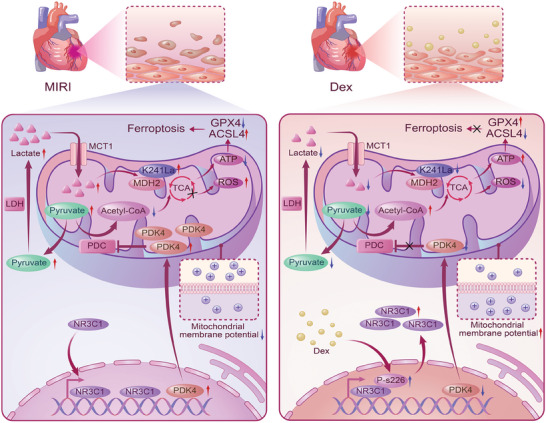
Schematic diagram showing the mechanism by which Dex alleviates myocardial ischemia‐reperfusion injury. Dex lowers lactate level and downregulates MDH2 lactylation to improve mitochondrial function and inhibit ferroptosis, thereby alleviating MIRI. This process involves the promotion of the phosphorylation and nuclear export of NR3C1 by Dex, which suppresses PDK4 expression and regulates metabolic reprogramming.

Our research indicated that MIRI was associated with metabolic reprogramming, leading to elevated lactate levels. Dex downregulated PDK4 by upregulating NR3C1 phosphorylation, leading to a decrease in lactate production and a reduction in MDH2 K241 lactylation, thus resulting in decreased ferroptosis and improved mitochondrial function, ultimately alleviating MIRI. This study provided a novel perspective on the mechanism of MIRI by exploring metabolic reprogramming and nucleus‐mitochondria communication, and identified potential therapeutic interventions for treatment of MIRI (**Figure** **8**).

This study found that Dex can protect against MIRI by mitigating ferroptosis in cardiomyocytes. While cardiomyocytes are crucial for cardiac function and structure,^[^ ^34^, ^35^ ^]^ previous research also indicated that Dex can affect the functionality of endothelial and immune cells, such as altering endothelial permeability and macrophage activation.^[^ ^36^, ^37^ ^]^ Moreover, evidence suggests that the functions of endothelial and immune cells are closely related to myocardial injury.^[^ ^36^, ^38^ ^]^ Therefore, Dex may confer cardioprotective effects by acting directly on cardiomyocytes or indirectly influencing other cell types. To gain a comprehensive understanding of Dex's mechanisms, future research should explore its effects on various cell types to better elucidate the pathogenic mechanisms of MIRI.

Ferroptosis is a form of non‐apoptotic cell death characterized by iron‐dependent lipid peroxidation and dysregulation of antioxidant systems.^[^ ^39^ ^]^ The discovery of ferroptosis has offered new perspectives on the pathological mechanisms of various diseases, including neurodegenerative diseases, tumors, and a range of inflammatory conditions.^[^ ^40^, ^41^, ^42^ ^]^ Mitochondria, the central hub of cellular energy metabolism, produce a large amount of ROS during dysfunction, leading to lipid peroxidation and consequent ferroptosis.^[^ ^20^, ^43^ ^]^ Recent studies have identified the critical role of ferroptosis in MIRI. Cai et al.^[^ ^35^ ^]^ discovered that apoptosis and necrosis occurred in the early stages of I/R injury, whereas ferroptosis predominated during extended reperfusion periods. Ma et al.^[^ ^44^ ^]^found that ischemia triggered specific redox reactions of polyunsaturated fatty acid (PUFA)‐phospholipids in cardiomyocytes, serving as the initial signal for severe oxidative damage during reperfusion. These findings suggested that regulating ferroptosis could potentially mitigate MIRI. This study indicated that Dex might reduce MIRI by inhibiting ferroptosis through metabolic reprogramming, offering significant insights for future MIRI treatment.

Lactate is a product of glycolysis and serves as an important regulator of cellular metabolism. Lactylation is the modification of lysine residues by lactate molecules.^[^ ^11^ ^]^ Researches indicated that lactylation might regulate cellular physiological functions by altering the stability and activity of protein, and interacting with other molecules.^[^ ^45^, ^46^ ^]^ Various studies showed that lactylation was prevalent across different cell types and played significant roles in transcriptional regulation, cell signaling, energy metabolism, and organ dysfunction.^[^ ^47^, ^48^ ^]^ Downregulation of α‐Myosin Heavy Chain (α‐MHC) lactylation could lead to structural and functional damage to the heart, exacerbating heart failure.^[^ ^49^ ^]^ Fis1 lactylation could worsen sepsis‐induced acute kidney injury (SAKI), while reducing lactate levels and Fis1 lactylation could alleviate SAKI.^[^ ^21^ ^]^ This study found that lactate impaired mitochondrial function in cardiomyocytes through the upregulation of MDH2 lactylation, consequently leading to ferroptosis and contributing to MIRI. Interestingly, our metabolomics revealed an upregulation of both L‐lactate and D‐lactate in MIRI. The latest research suggested that lysine L‐lactylation was the dominant isomer.^[^ ^50^ ^]^ However, further research is needed to determine which specific form of lactylation plays a primary role in MIRI.

MDH2 is a critical enzyme located in the mitochondrial matrix to catalyze the reversible conversion between malate and oxaloacetate, which is essential for tricarboxylic acid (TCA) cycle^[^ ^51^ ^]^ Deficiency or malfunction of MDH2 can lead to the accumulation of intermediate metabolites in the TCA cycle, thereby affecting cellular energy supply.^[^ ^52^ ^]^ Additionally, MDH2 plays a crucial role in maintaining redox balance by regulating the NADH/NAD + ratio.^[^ ^53^ ^]^ MDH2 dysfunction leads to a reduction in NADH production, thereby impairing the efficiency of the electron transport chain and increasing ROS production.^[^ ^54^, ^55^ ^]^ The latest research found that the post‐translational modifications of the MDH2 could affect its function.^[^ ^22^ ^]^ A study discovered that MDH2 palmitoylation can activate mitochondrial function, while our research indicated that MDH2 lactylation was associated with MIRI, potentially by regulating mitochondrial function and ferroptosis.

PDK4 plays a crucial role in lactate metabolism. By inhibiting the activity of pyruvate dehydrogenase (PDH), PDK4 prevents the conversion of pyruvate to acetyl‐CoA and promotes lactate production.^[^ ^56^, ^57^ ^]^ Recent studies showed that upregulation of PDK4 was closely associated with cardiovascular dysfunction. Research has indicated a correlation between the expression of PDK4 and the phenotype of more rupture‐prone plaques, suggesting that inhibiting PDK could reduce vascular inflammation and atherosclerosis.^[^ ^58^ ^]^ Another study also found that inhibiting PDK4 might have cardioprotective effects, as PDK4 inhibitors potentially enhanced bioenergetics by activating the TCA cycle, thereby improving ejection fraction in failing hearts.^[^ ^59^ ^]^ Additionally, PDK4 was identified as a significant target for treating sepsis‐induced cardiomyopathy.^[^ ^60^ ^]^ Our study also found that PDK4 upregulation was related to MIRI. Dex downregulated PDK4 and thus inhibited lactate production, which provided a protective effect for MIRI.

NR3C1/glucocorticoid receptor functions as a transcription factor and participates in the regulation of complex biological processes such as stress response, metabolism, immune response, and inflammation.^[^ ^61^, ^62^ ^]^ In its inactive state, NR3C1 is typically located in the cytoplasm and bound to regulatory chaperone proteins like HSP90. Upon activation, NR3C1 is released from the chaperone protein complex and translocate to the nucleus, where it binds to specific sequences known as Glucocorticoid Response Elements (GREs), thereby regulating the transcription of target genes.^[^ ^31^, ^32^ ^]^ NR3C1 can also translocate back to the cytoplasm via a nuclear export. This intracellular cycling is crucial for maintaining the dynamic equilibrium of NR3C1's diverse physiological functions.^[^ ^63^, ^64^ ^]^ Phosphorylation significantly influences NR3C1's function. Depending on the phosphorylation site, NR3C1's stability, transcriptional activity, and subcellular localization can be differentially affected.^[^ ^65^ ^]^ Phosphorylation at the Ser211 site enhances NR3C1's transcriptional activity,^[^ ^66^ ^]^ whereas phosphorylation at the Ser226 site promotes nuclear export of NR3C1.^[^ ^33^, ^64^, ^67^ ^]^ Additionally, phosphorylation at various sites influences NR3C1's ability to bind to different target genes^[^ ^33^ ^]^; phosphorylation at the Ser226 site is also associated with inflammation.^[^ ^68^ ^]^ In this study, inhibiting phosphorylation of NR3C1 by S226A mutation increased mitochondrial ferrous ion and lipid peroxidation, weakening the protective effect of Dex on cardiomyocytes in I/R rats.

## 4 Conclusion

In summary, this study demonstrated that Dex could protect cardiac function by regulating NR3C1 phosphorylation to upregulate PDK4 and inhibit lactate production, offering a new therapeutic perspective for MIRI.

## 5 Experimental Section

### Ethical Review

The human study was approved by the Ethics Committee of the Army Medical University (No. 2021–119) and was registered with the Chinese Clinical Trial Registry (ChiCTR2200061798). All participants provided written informed consents prior to inclusion in the study. The animal experiments adhered to the guidelines of Reporting of In Vivo Experiments (ARRIVE). The experimental protocol was approved by the Laboratory Animal Welfare and Ethics Committee of the Army Medical University (No. AMUWEC20224867).

### Reagents

Dex was purchased from Hengrui (Jiangsu, China). Antibody for β‐actin (ab8226) was purchased from Abcam (Cambridge, MA, USA). Antibody for ACSL4 (GTX635616) was purchased from GeneTEX (San Antonio, TX, USA). Antibodies for GPX4 (52455), Phospho‐NR3C1 (Ser226) (97285), and NR3C1 (12041) were purchased from Cell Signaling Technology (Danvers, Massachusetts, USA). Antibodies for MDH2(A20968), PDK4 (A13337), AR (A19611), PGR (A19697), and GAPDH(AC001) were purchased from ABclonal (Wuhan, China). Antibodies for Lactyl‐lysine (PTM‐1401RM) and Histone H3 (PTM‐1002RM) were obtained from PTM Biolabs (Hangzhou, China). DCFH‐DA (S0033M), JC‐1 (C2003S), MDA (S0131M), Glutathione (S0053), and ATP (S0026) detection kits were purchased from Beyotime Biotechnology (Shanghai, China). Lactate assay kit (BC2230) was purchased from Solarbio (Beijing, China). MitoBright‐Deep Red (MT12) and Mito‐FerroGreen (M489) were purchased from Dojindo (Kyushu, Japan). All inhibitors were sourced from MedChemExpress (Monmouth, NJ, America) including Ferrostatin‐1 (HY‐100579), Necrostatin‐1 (HY‐15760), Emricasan (HY‐10396), and 3‐Methyladenine (HY‐19312). Adenoviral vectors for PDK4 overexpression (PDK4OE) and NR3C1 mutation (S226A) were generated by Genechem Technology (Shanghai, China). Lentiviral vectors for MDH2 K241 mutations (K241T and K241R), Adeno‐associated viruses for MDH2 K241 mutations, and NR3C1 mutation (S226A) were generated by Obio life Technology (Shanghai, China). All other chemicals were purchased from Sigma–Aldrich (St. Louis, MO, USA) unless specifically mentioned.

### Study Design and Population Recruitment

The study recruited 60 patients diagnosed with heart valve disease who underwent valve replacement surgery (including annuloplasty) with cardiopulmonary bypass (CPB) in the Cardiovascular Surgery Department of Daping Hospital, Army Medical University, from November 2021 to January 2023. The inclusion and exclusion criteria for patients are presented in Table S1 (Supporting Information). Using a random number method, the 60 patients were randomly divided into the Dex group and the control group (Con group) in a 1:1 ratio. During the entire trial period, the participants were blind to the treatments.

### Anesthesia and Administration Methods

All patients were routinely monitored for electrocardiography, blood pressure, oxygen saturation, and anesthetic depth using bispectral index (BIS). Pure oxygen was administered through a face mask. A peripheral intravenous route was established, and radial artery puncture was performed for blood pressure measurement and blood gas analysis. In the Dex group, a loading dose (0.5 µg kg^−1^) of Dex was administered over 10 min before anesthesia induction, followed by continuous infusion at 0.5 µg kg^−1^h^−1^ until the end of surgery. Postoperatively, patients were transferred to the ICU, where Dex infusion was continued at 0.3 µg kg^−1^h^−1^ for 6 h. In the Con group, an equal volume and rate of 0.9% saline solution were infused instead.

Induction of anesthesia was achieved with intravenous administration of midazolam (0.05–0.15 mg kg^−1^), sufentanil (0.5–1 µg kg^−1^), etomidate (0.15–0.3 mg kg^−1^), and rocuronium bromide (0.6–0.8 mg kg^−1^). Upon successful induction, endotracheal intubation was performed, followed by mechanical ventilation. Ventilation was controlled in a volume‐control mode, with tidal volumes of 8–10 mL kg^−1^ and a frequency of 14–16 breaths per min. The inspiratory to expiratory ratio was set at 1:2, aiming to maintain an end‐tidal CO_2_ pressure (ETCO_2_) between 30 and 40 mmHg. Anesthesia was maintained with continuous infusions of midazolam (0.02–0.1 mg kg^−1^h^−1^), sufentanil (0.5–1.5 µg kg^−1^h^−1^), rocuronium bromide (0.3–0.6 mg kg^−1^h^−1^), and sevoflurane (0.5–2%). Additionally, nitroglycerin (0.01–0.5 µg kg^−1^ min^−1^) and dopamine (0.1–15 µg kg^−1^min^−1^) were continuously infused intraoperatively. All patients underwent internal jugular vein catheterization under ultrasound guidance for central venous pressure (CVP) measurement and were monitored using transesophageal echocardiography (TEE).

### Collection of Blood Samples and Extraction of Metabolites

Fasting venous blood (5 mL) was collected in the morning on the day of surgery and on the third postoperative day. The collected blood was left to stand at room temperature in a centrifuge tube for 1 h to coagulate. Then the blood was centrifuged at 3000 rpm for 10 min, and the supernatant was transferred to a clean centrifuge tube, flowed by another centrifugation at 12 000 rpm and 4 °C for 10 min. About 0.2 mL resultant supernatant was transferred into 1.5 mL centrifuge tubes and stored at −80 °C. For analysis, 50 µL of the sample with 200 µL of extraction solution [methanol: acetonitrile = 1:1 (V/V), containing isotope‐labeled internal standards] was added into an EP tube, and mixed by vortexing for 30 s. The mixture was sonicated for 10 min in an ice‐water bath, left to stand at −40 °C for 1 h, and then centrifuged at 12 000 rpm and 4 °C (centrifugal force 13 800 × g, radius 8.6 cm) for 15 min. An equal amount of supernatant from each sample was pooled, mixed uniformly, and used as a quality control (QC) sample for analysis.

### Metabolomics Analysis

A Vanquish ultra‐high‐performance liquid chromatography (UHPLC) system (Thermo Fisher Scientific) was employed, using a Waters ACQUITY UPLC BEH Amide column (2.1 mm × 100 mm, 1.7 µm) for the separation of target compounds. The mobile phase consisted of a water phase (Phase A) and an acetonitrile phase (Phase B), in which Phase A contained 25 mmol L^−1^ ammonium acetate and 25 mmol L^−1^ ammonium hydroxide. The sample tray was maintained at 4 °C, and the injection volume was 3 µL. Raw data were converted to the mzXML format using ProteoWizard software. Peak identification, extraction, alignment, and integration were then performed using a custom‐developed R package (based on XCMS). Compound annotation and matching were conducted using the BiotreeDB (V2.1) in‐house mass spectrometry database, with the cutoff score set at 0.3. Orthogonal partial least squares‐discriminant analysis (OPLS‐DA) was utilized, and compounds with a variable importance in projection (VIP) score >1.0 and P‐value < 0.05 were selected as differential metabolites.^[^ ^69^ ^]^

### In Vivo Myocardial Ischemia/Reperfusion (I/R) Model

Adult male and female Sprague Dawley rats (200–220 g) were obtained from the Animal Center of the Research Institute of Surgery. They were housed in a facility with filtered positive‐pressure ventilation, food, and water ad libitum, at 23—25 °C and 40–70% humidity. The surgical area was shaved and disinfected. After the rats were anesthetized, an oblique incision was made along the lower edge of the left pectoralis major muscle. The muscle was separated through the third intercostal space, and the intercostal space was penetrated to expose the heart. Ligation was performed at the terminal end of the vein leading to the heart's apex. A needle was inserted 2–3 mm above and 1–2 mm left to the bifurcation point and thread out from the right side, and a 6‐0 suture was used for ligation, leaving a 3–4 cm tail outside the thoracic cavity. After ligation, the heart was returned to the thoracic cavity, and air was expelled before closing. After 45 min of ischemia, the ligation was removed to restore blood flow. Following 24 h of reperfusion, the heart was harvested. Rats in the Dex group received Dex (10 µg kg^−1^)^[^ ^20^ ^]^ intraperitoneally 30 min before surgery and another dose 12 h after reperfusion. In the Dex + Lac group, 1 g kg^−1^ sodium lactate (Sigma, 71718)^[^ ^21^ ^]^ was intraperitoneally injected 10 min before Dex administration.

### TTC‐Evans Staining of Rat Cardiac Tissue

After the rats were anesthetized, the ischemic site of heart was re‐ligated, and 2 mL of 3% Evans Blue dye was injected via the jugular vein. The hearts were harvested and stored at −80 °C for 5 min before being sectioned into five slices below the ligature line. The slices were then immersed in 1% TTC buffer (pH 7.4) and incubated at 37 °C for 5 min. After staining, photographs were taken, and interested areas were measured using Image‐Pro Plus 6.0 software. Normal tissue was stained deep blue, ischemic but non‐infarcted tissue was stained red, and infarcted tissue was stained pale. The ratio of area at risk (AAR, red and pale tissue) to the left ventricular area (LV) (%) represented the size of the ischemic region, whereas the ratio of infarct area (IA, pale tissue) to AAR (%) represented the size of the infarcted region.^[^ ^70^ ^]^

### Echocardiography for Measuring Rat LVEF

Isoflurane was administered to anesthetize the rats, ensuring a stable physiological state throughout the testing process and preventing any impact on the cardiac function results. Subsequently, the rats’ chest hair was shaved, and the Mindray S7 SCI high‐frequency color Doppler ultrasound system with a 30 MHz high‐frequency ultrasound probe was employed to obtain ultrasound images of the left ventricle from the parasternal long‐axis view. The internal diameter changes of the left ventricle during the cardiac cycle were measured using the obtained M‐mode ultrasound images, and the LVEF was automatically calculated by software.

### Isolation of Rat Cardiomyocytes

Isolation of cardiomyocytes was conducted as previously described.^[^ ^71^ ^]^ Using the Langendorff method, rat hearts were perfused with calcium‐free Tyrode's solution for 5 min, followed by a digestive solution until the hearts became enlarged and softened. The hearts were then excised, and the atrial region was removed under the pre‐cooled KB solution. The myocardial tissue was minced, triturated, and filtered to prepare single cardiomyocyte. A concentration gradient method was used for calcium addition, and the cells were finally resuspended in 1 mL of Tyrode's solution containing 1 mm Ca^2^⁺.

### Measurement of Cardiomyocytes Contraction Function

To conduct the experiment, 1.8 mm Ca^2^⁺ Tyrode solution was added to the stimulation bath of the microtension monitoring device (Ionoptix, USA) to submerge the stimulating electrode. Subsequently, 100 µL of the above cardiomyocytes suspension containing Ca^2^⁺ was placed into the stimulation bath. The cardiomyocytes were then stimulated with a voltage of 15 V at a frequency of 1 Hz continuously for 5 min to stabilize the contraction amplitude and rhythm. Following stabilization, the length of sarcomere was recorded during 10–20 stable contractions. The IonWizard software was utilized to integrate these 10–20 contraction data points into an average contraction curve, with time as the x‐axis and normalized sarcomere length as the y‐axis (normalized sarcomere length = sarcomere length/resting sarcomere length). The y‐axis reflected cardiomyocytes contraction amplitude, and the slope represented the contraction speed.

### Cell Culture

Rat cardiomyocyte H9c2 cells were procured from the Cell Bank of the Chinese Academy of Sciences in Shanghai. The cells were cultured in Dulbecco's Modified Eagle Medium (DMEM, Gibco, NY, USA) supplemented with 10% fetal bovine serum and 1% penicillin‐streptomycin solution. The culture conditions were maintained at 37 °C with 5% CO2 and 95% humidity.

### Cell Oxygen‐Glucose Deprivation/Reoxygenation (OGD/R) Model and Treatment

H9c2 cells were cultured in a hypoxia chamber with hypoxia‐equilibrated medium (serum‐free, glucose‐free DMEM) containing a 95% N_2_ and 5% CO_2_ gas mixture for 6 h. Subsequently, the cells were re‐oxygenated with maintenance medium for 12 h to induce OGD/R injury. To investigate the protective role of Dex against OGD/R‐induced injury in H9c2 cardiomyocytes, the cells were pretreated with Dex (1 µm) for 30 min before exposure to OGD/R.^[^ ^20^ ^]^

### Lentiviral Construction and Cell Transfections

A lentiviral packaging system was employed to overexpress the MDH2 (K241T) and MDH2 (K241R) variants. The vectors pSLenti‐SFH‐P2A‐Puro‐CMV‐MDH2 (K241T)‐WPRE and pSLenti‐SFH‐P2A‐Puro‐CMV‐MDH2 (K241R)‐WPRE were transfected into H9c2 cell lines following the manufacturer's protocols to establish cell lines overexpressing MDH2 mutations. Transduced cells were selected using 2 µg mL^−1^ puromycin (EZ2811D376, BioFroxx, Germany) and maintained in a medium containing 1 µg mL^−1^ puromycin.

### The Detection of Lactylation Proteomics

Samples were retrieved from −80 °C and mixed with 4 volumes of lysis buffer containing 8 m urea, 1% protease inhibitor, 3 µm TSA, and 50 mm NAM, followed by sonication for lysis. The mixture was centrifuged at 12 000 g for 10 min at 4 °C to remove cell debris, and the supernatant was collected. Protein concentration was determined using the BCA assay. The equal amounts of samples were treated with 20% TCA and allowed to precipitated at 4 °C for 2 h. The precipitate was centrifuged at 4500 g for 5 min, and the supernatant was discarded, leaving the pellet being washed with cold acetone, dried and then resuspended in 200 mm TEAB before sonication. Trypsin was added at a 1:50 (protease: protein) ratio for overnight digestion. Reduction was performed with 5 mm DTT at 56 °C for 30 min, followed by alkylation with 11 mm IAA in the dark for 15 min. The peptides were dissolved in IP buffer (100 mm NaCl, 1 mm EDTA, 50 mm Tris‐HCl, 0.5% NP‐40, pH 8.0) and incubated overnight at 4 °C with lactylated resin on a rotary shaker. The resin was washed four times with IP buffer, twice with deionized water, and peptides were eluted three times with 0.1% trifluoroacetic acid. The eluates were collected and vacuum‐dried. Peptides were desalted using C18 ZipTips, vacuum‐dried again, and analyzed by liquid chromatography‐mass spectrometry (LC‐MS). Peptides were dissolved in mobile phase A and separated using the NanoElute ultra‐high‐performance liquid chromatography system. The separated peptides were injected into a capillary ion source and analyzed by timsTOF Pro mass spectrometry. The ion source voltage was set to 1.6 kV, and both precursor and fragment ions were detected with high‐resolution time‐of‐flight (TOF). The MS/MS scanning range was 100–1700 m z^−1^, and data acquisition was performed in PASEF mode. After each primary mass spectrometry scan, 10 PASEF scans were conducted. Precursors with charge states of 0–5 were dynamically excluded for 30 s to avoid repeated scans.

### Mitochondrial Oxygen Consumption Rate Detection

The oxygen consumption rate (OCR) was measured using a 24‐well XFe plate (Seahorse, Agilent Cell Analysis Technology, USA). Cells were seeded at a density of 1 × 10^4^ per well and allowed to settle naturally for 1 h in a sterile environment. The cell plate was then incubated overnight to facilitate cell adhesion. Upon reaching 70–80% confluence, cells were subjected to the OGD/R model construction. Before detection, cells were cultured for 50 min in a basal assay medium containing 2.5 µm glucose and 2 mm glutamine. Sequentially added of 2 µm oligomycin, 1 µm FCCP, and 0.5 µm rotenone/antimycin A. The OCR was subsequently measured using an extracellular flux analyzer under mitochondrial stress test conditions.

### Glycolysis Rate Detection

Cells were seeded into a Seahorse XFe24 cell culture plate at a density of 1 × 10^4^ cells per well. After allowing the cells to settle naturally for 1 h, the plate was incubated overnight in a 37 °C CO_2_ incubator. The Seahorse assay medium was then prepared, which primarily consisted of a base medium with added substrates like glucose, glutamine, and pyruvate. The cell culture medium was replaced with the assay medium, and the plate was incubated for 1 h in a 37 °C incubator without CO_2_. Diluted drugs (Rot/AA and 2‐DG) were added to the respective wells of the probe plate. The XF experiment was conducted using the Seahorse XF Analyzer, and the data were analyzed with the Wave software and report generator.

### Lipid Peroxidation Detection

Cells were incubated with a 10 µm lipid peroxidation kit (C10445; Invitrogen) for 30 min at 37 °C. FITC fluorescence was detected at excitation/emission wavelengths of 488/510 nm, and TRITC fluorescence was detected at excitation/emission wavelengths of 581/591 nm using confocal microscopy (Leica SP5, Germany). The ratio of fluorescence intensities at 510 to 590 nm served as an indicator of lipid peroxidation in cells.

### Immunofluorescence Staining

Cells were seeded into a confocal microscopy chamber and incubated with MitoTracker (1:10 000) at 37 °C for 30 min. Mitochondrial morphology was then observed using a laser confocal microscope (Leica SP5, Germany). Mitochondrial length was analyzed using the Mitochondrial Network Analysis (MiNA) toolset in the ImageJ software. Additionally, the cells were incubated with DCFH‐DA (10 µm) and JC‐1 (1:1000) at 37 °C for 30 min. The average fluorescence intensity of intracellular ROS and the ratio of red to green fluorescence of the mitochondrial membrane potential were subsequently calculated with ImageJ.

### Statistical Analysis

Statistical analyses were performed using SPSS (version 20.0). Results are presented as mean ± standard deviation (SD). Animal experiments were conducted independently at least six rats each group, and cell experiments were repeated at least three times. Differences between two groups were analyzed with independent t‐tests. For more than two groups, one‐way ANOVA followed by Tukey's post‐hoc test was used. Additional comparisons employed the Student‐Newman‐Keuls (SNK) or least significant difference (LSD) tests. Statistical significance was set at P < 0.05.

### Ethics Approval and Consent to Participate

The human study was approved by the Ethics Committee of the Army Medical University (No. 2021–119) and was registered by the Chinese Clinical Trial Registry (ChiCTR2200061798). All the participants provided written informed consents before inclusion in this study. The animal experiments adhered to the guidelines of Reporting of In Vivo Experiments (ARRIVE). The experiment protocol was approved by Laboratory Animal Welfare and Ethics Committee of the Army Medical University (No. AMUWEC20224867).

## Conflict of Interest

The authors declare no conflict of interest.

## Author Contributions

S.H., H.Y., and Z.G.Z. contributed equally to this work. S.H., H.Y., and Z.G.Z. performed the experiments and drafted the manuscript. S.H. and D.Y.X. conducted animal experiments and analyzed the data. W.Y.Y. and L.Q.H. participated in bioinformatic analysis. S.H., Z.D.Y., L.L.M., and L.T. designed the whole project. S.H., Z.D.Y., L.T., L.L.M., and M.Q.X. were responsible for manuscript editing and revision, and they provided scientific research funding support. All authors read and approved the final manuscript.

## Supporting information



Supporting Information

## Data Availability

The data that support the findings of this study are available from the corresponding author upon reasonable request.
