# Supplementary material for: Dexmedetomidine Ameliorates Myocardial Ischemia‐Reperfusion Injury by Inhibiting MDH2 Lactylation via Regulating Metabolic Reprogramming
Source: Adv Sci (Weinh). 2024 Oct 28;11(48):2409499. doi: 10.1002/advs.202409499 (PMC11672254; doi:10.1002/advs.202409499)
Supplement: Supplementary file 1 — Supporting Information [file ADVS-11-2409499-s001.docx]

**Table S1.** The inclusion and exclusion criteria of patients.

| Inclusion criteria | 1. Diagnosed with heart valve disease and underwent valve replacement surgery with cardiopulmonary bypass. 2. The patient provided informed consent and participated voluntarily. 3. 30-65 years old (inclusive of 65 years old). 4. American Society of Anesthesiologists (ASA) rating II-III. 5. Record general medical conditions. |
| --- | --- |
| Exclusion criteria | 1. Age > 65 years or ≤ 30 years. 2. History of cognitive dysfunction or having undergone surgeries or medications affecting cognitive function. 3. Suffering from diabetes, hyperlipidemia, and other metabolic diseases. 4. History of central nervous system diseases (stroke, schizophrenia, epilepsy, and Parkinson’s). 5. Use of other experimental drugs or participation in other clinical trials within 3 months before the surgery. 6. Patients with hearing, vision, or language disabilities, or those who are unable to write. 7. Alcohol or drug dependence. 8. Arrhythmias such as sick sinus syndrome or severe bradycardia. 9. Patients with a left ventricular ejection fraction (LVEF) of less than 35%. 10. Patient or family refusal to be involved in the study. |

**Table S2.** Baseline characteristics of the two groups of patients.

| **Characteristics** |  | **Con** | **Dex** | **p-Value** |
| --- | --- | --- | --- | --- |
|  |  | **(n=30)** | **(n=30)** |  |
| Age, yrs | Mean | 57.2 | 59.7 | 0.162 |
|  | SD | 6.7 | 7.1 |  |
| Gender, n (%) | Female | 13 (43.3) | 15 (50.0) | 0.605 |
|  | Male | 17 (56.7) | 15 (50.0) |  |
| ASA classification, n (%) | II | 11 (36.7) | 10 (33.3) | 0.787 |
|  | III | 19 (63.3) | 20 (66.7) |  |
| Surgery time, min | Mean | 377.2 | 386.2 | 0.365 |
|  | SD | 38.4 | 37.9 |  |
| CPB time, min | Mean | 261.3 | 272.2 | 0.256 |
|  | SD | 37.1 | 36.9 |  |
| Conventional medication (%) |  |  |  |  |
| Metoprolol | Yes | 3(10.0) | 2(6.7) | 0.640 |
|  | No | 27(90.0) | 28(93.3) |  |
| Furosemide | Yes | 2(6.7) | 1(3.3) | 0.554 |
|  | No | 28(93.3) | 29(96.7) |  |
| Amlodipine | Yes | 2(6.7) | 3(10.0) | 0.640 |
|  | No | 28(93.3) | 27(90.0) |  |
| Metformin | Yes | 3(10.0) | 2(6.7) | 0.640 |
|  | No | 27(90.0) | 28(93.3) |  |
| Aspirin | Yes | 3(10.0) | 3(10.0) | 0.966 |
|  | No | 27(90.0) | 27(90.0) |  |

**Figure S1.**


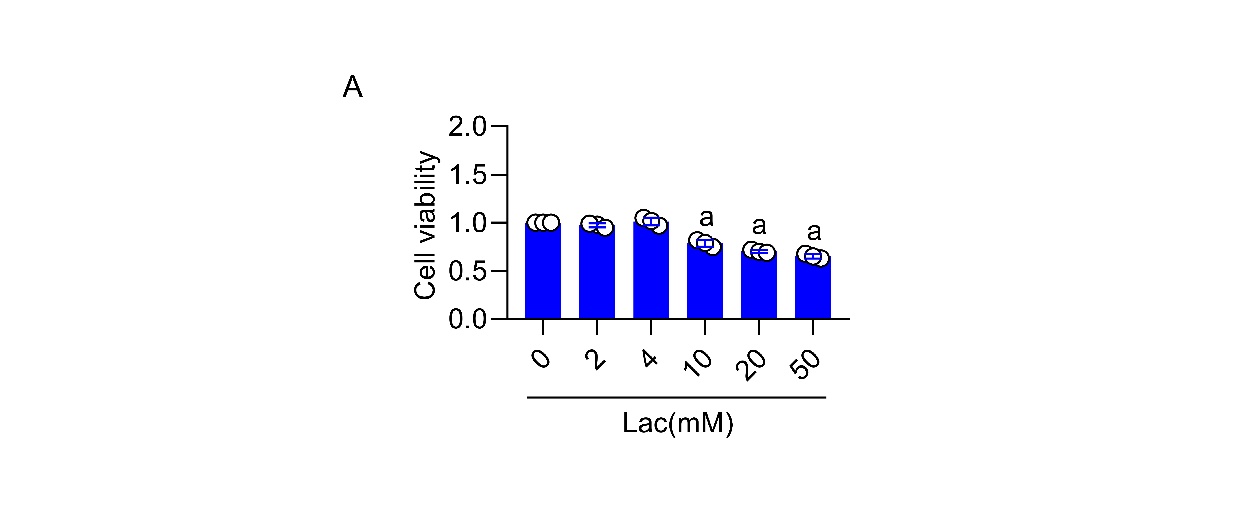


(A) Effects of different concentrations (0~50 mM) of lactate on the viability of H9c2 cells (n=3 independent experiments). a: p<0.05, as compared with the 0 mM group.

**Figure S2.**


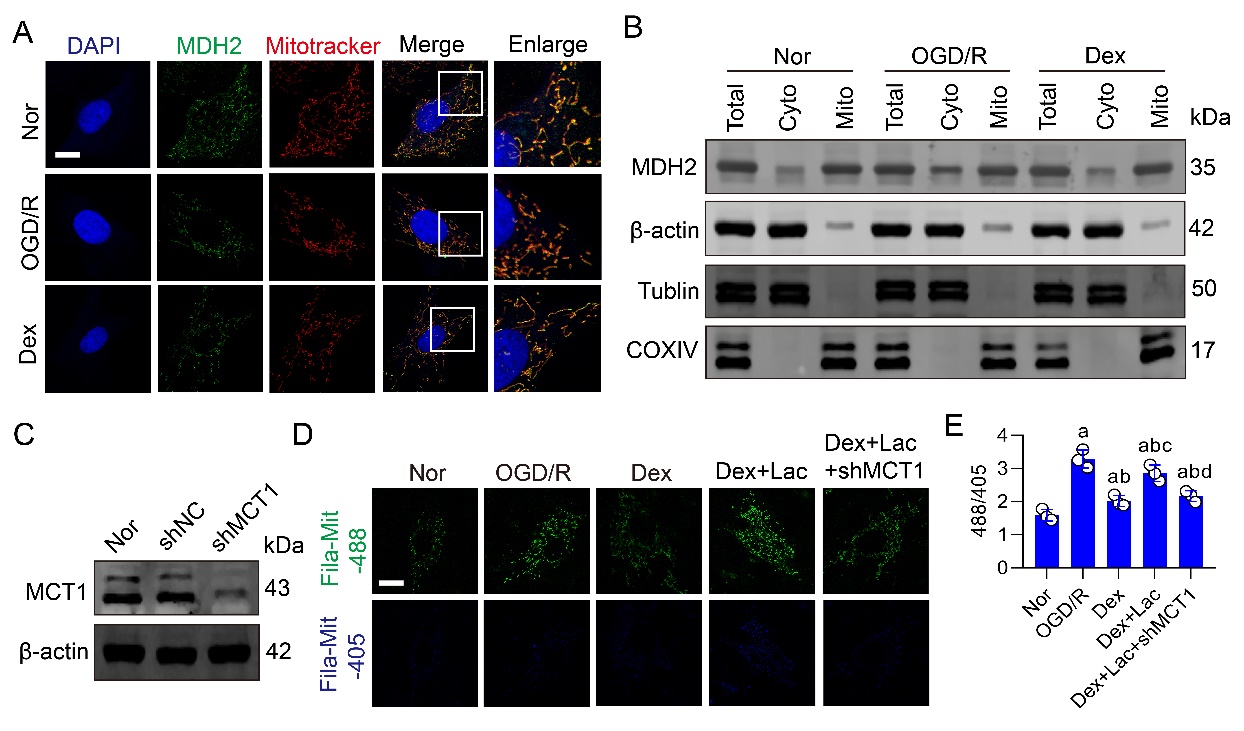


(A) Immunofluorescence was used to detect the colocalization of MDH2 and mitochondria in different groups of H9c2 cells. MDH2 was labeled with green fluorescence, while mitochondria were stained with red fluorescence (bar=10μm) (n=3 independent experiments). (B) The expressions of MDH2 in total, cytoplasmic, and mitochondrial fractions of H9c2 cells were assessed. β-actin, Tubulin, and COX IV were served as internal references for the total, cytoplasmic, and mitochondrial fractions, respectively (n=3 independent experiments). (C) The efficiency of MCT1 interference was validated (n=3 independent experiments). (D-E) The impact of different treatments on mitochondrial lactate level in H9c2 cells was evaluated by the mitochondrial lactate probe Fila-Mit. Imaging was performed using the 488 and 405 channels, with the emission wavelength set to 500-550 nm. The mitochondrial lactate level was reflected by the fluorescence intensity ratio of 488/405 (bar=10μm) (n=3 independent experiments). a: p<0.05, compared with the Nor group; b: p<0.05, compared with the OGD/R group; c: p<0.05, compared with the Dex group; d: p<0.05, compared with the Dex+Lac group. Nor: normal group; OGD/R: OGD/R group; Dex: Dex-treated OGD/R group; Dex+Lac: Dex+Lac treated OGD/R group; Dex+Lac+shMCT1: Dex+Lac+shMCT1+OGD/R group.

**Figure S3.**


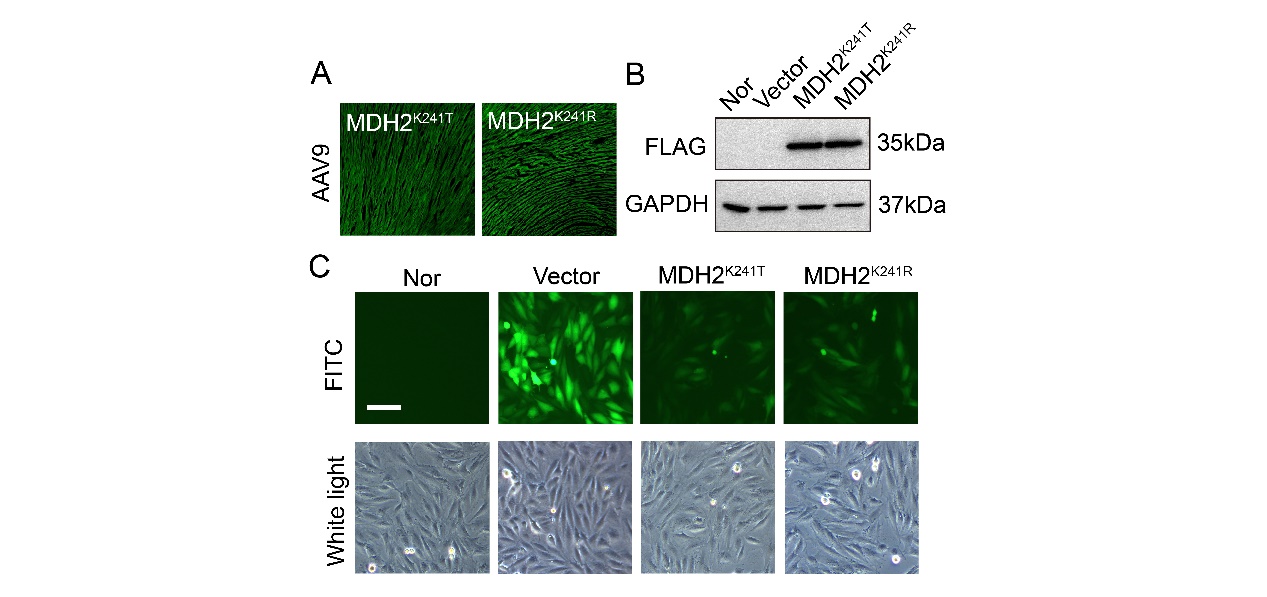


(A) Representative images show Adeno-associated viruses (AAVs) -infected rat myocardial tissue. AAVs with the heart-specific promoter cTNT were generated to introduce the point mutations in MDH2, including pcAAV-cTNT-MDH2 (K241T)-3xFLAG-P2A-GdGreen-WPRE and pcAAV-cTNT-MDH2 (K241R)-3xFLAG-P2A-GdGreen-WPRE. The rats were injected with the corresponding AAVs through the tail vein (1.5×10¹² vg per rat) 28 days before I/R modeling. (B) WB detection of lentiviral transduction efficiency of MDH2 mutants (n=3 independent experiments). (C) Fluorescence images of lentiviral transduction of MDH2 mutants at 72h post-transduction (bar = 50μm).

**Figure S4.**


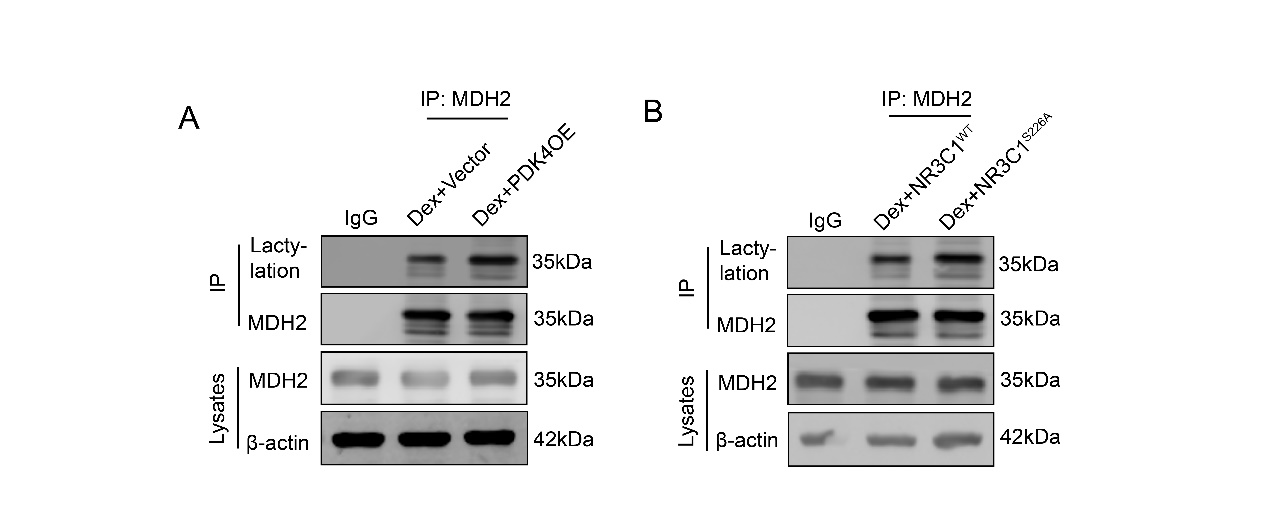


(A) Immunoprecipitation analysis of the effect of PDK4OE on the lactylation level of MDH2 (n=3 independent experiments). (B) Immunoprecipitation analysis of the effect of NR3C1^S226A^ mutation on the lactylation level of MDH2 (n=3 independent experiments).
